# Effective processing and evaluation of chemical imaging data with respect to morphological features of the zebrafish embryo

**DOI:** 10.1007/s00216-020-03131-4

**Published:** 2021-02-01

**Authors:** Katharina Halbach, Timothy Holbrook, Thorsten Reemtsma, Stephan Wagner

**Affiliations:** 1 Department of Analytical Chemistry, Helmholtz Centre for Environmental Research - UFZ, 04318 Leipzig, Germany; 2 Institute of Analytical Chemistry, University of Leipzig, 04103 Leipzig, Germany; 3 Present Address: Institute for Water and Energy Management, University of Applied Sciences Hof, 95028 Hof, Germany

**Keywords:** MS imaging, Toxicokinetics, Biological samples, Cluster analysis, ICP-ToF-MS, Organ

## Abstract

A workflow was developed and implemented in a software tool for the automated combination of spatially resolved laser ablation-inductively coupled plasma-mass spectrometry (LA-ICP-MS) data and data on the morphology of the biological tissue. Making use of a recently published biological annotation software, FishImager automatically assigns the biological feature as regions of interest (ROIs) and overlays them with the quantitative LA-ICP-MS data. Furthermore, statistical tools including cluster algorithms can be applied to the elemental intensity data and directly compared with the ROIs. This is effectively visualized in heatmaps. This allows gaining statistical significance on distribution and co-localization patterns. Finally, the biological functions of the assigned ROIs can then be easily linked with elemental distributions. We demonstrate the versatility of FishImager with quantitative LA-ICP-MS data of the zebrafish embryo tissue. The distribution of natural elements and xenobiotics is analyzed and discussed. With the help of FishImager, it was possible to identify compartments affected by toxicity effects or biological mechanisms to eliminate the xenobiotic. The presented workflow can be used for clinical and ecotoxicological testing, for example. Ultimately, it is a tool to simplify and reproduce interpretations of imaging LA-ICP-MS data in many applications.

**Supplementary Information:**

The online version contains supplementary material available at 10.1007/s00216-020-03131-4.

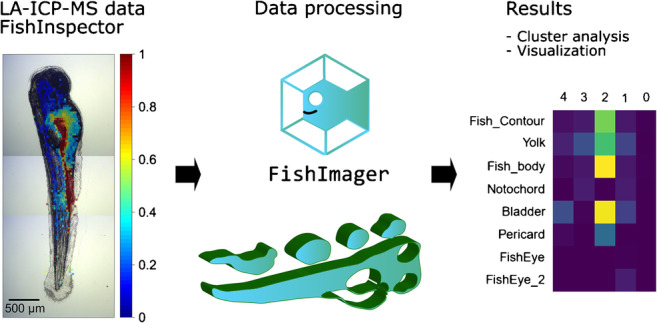

## Introduction

The zebrafish embryo (ZFE, *Danio rerio*) is an important model organism and used in various research fields such as developmental biology, biomedical research, and (eco)toxicology [1]. Imaging of biological or toxicological processes within the ZFE is increasingly reported [2–6]. One versatile quantitative elemental imaging technique for biological tissues is laser ablation-inductively coupled plasma-mass spectrometry (LA-ICP-MS). It is applied in various applications, such as determining the distribution of anti-cancer compounds in tumor tissue and nanoparticles in organisms, or the (immune) response on environmental contaminants, as well as localization of X-ray contrast agents [3, 7–11]. Spatial resolution and sensitivity have been strongly improved, acquisition time has been decreased, and new quantification strategies have been developed over the past years [12–14]. Although there are numerous software tools to combine and visualize the spatial laser ablation coordinates with the ICP-MS data [15–17], data post-processing is still in its infancy. The interpretation of imaging data is often solely based on the visual comparison of a color-coded picture representing the intensity or quantified amount. This interpretation relies on the expert knowledge of the analyst [18]. Furthermore, the combination of LA-ICP-MS results with biological information (e.g., at the sub-organ level) is achieved with manual overlays of different images using several software products [10]. Yet, the LA-ICP-MS data set may allow more objective information to be discovered. According to available studies, image analysis software for LA-ICP-MS data shall be extended with:

(i) Quality controls like measurement uncertainty and validation parameters such as limit of detection and quantification, linearity of the calibration range, precision, and accuracy.
(ii) Tools for statistical analyses such as cluster analysis and principal component analysis. These have been recently included in the software HDIP (Hierarchical Data Format version 5-image processing) [19].
(iii) Algorithms to correlate spatial data from LA-ICP-MS and other imaging techniques.

Biological studies often require definitions of regions of interest (ROIs) to investigate research questions such as (co-)localization of chemicals/elements and toxicokinetic processes in certain tissue compartments. ROIs are often manually assigned in the data processing software to the LA-ICP-MS results and then compared [20, 21]. This, however, comes together with subjective identifications depending on the quality of the images and the expertise of the analyst and may be time-consuming. Automated identification of ROIs based on sample features (e.g., organs, body compartments) can be performed in application-specific software, but these results cannot be easily combined with the LA-ICP-MS data [17, 22]. The assignment might even be based on a different image other than that of the camera delivered with the laser ablation system, e.g., taken with a high-resolution microscope. The combination of spatially resolved information from different sources is still rarely reported due to the complexity of data transformation and missing software tools. However, the establishment of quantitative LA-ICP-MS image analysis for diagnostics in medicine, pharmacology studies, and (eco)toxicological studies requires an increased effort to generate reproducible data, to link them with data from other analyses, and with morphology and function, for example.

Here, we demonstrate the data post-processing software FishImager for the combination of results from an assessment of morphological features (biological ROIs) with chemical imaging data including statistical tools such as different cluster analysis algorithms. The automatically assigned biological ROIs can be linked with the quantitative LA-ICP-MS data (incl. LA-ICP-ToF-MS data) and be individually evaluated and compared with each other. For instance, quantified amounts or mean elemental intensities may be calculated for each ROI. The software allows applying different cluster algorithms, depending on the LA-ICP-MS data set structure, the biological tissue, and the research question. The assigned clusters can then be compared with the biological ROIs, e.g., overlapping areas and elemental content. The commercially available software Iolite and HDIP offer some statistical analyses and manual ROI drawings and investigations; however, importing and combining other biological information such as the morphological features is to our knowledge not supported [16, 19, 23]. The cluster algorithm k-means on LA-ICP-MS data was recently used to identify sub-organ regions [24]. While FishImager is not meant to replace these software, we wish to enhance the application of LA-ICP-MS data to biological and environmental research questions. FishImager is freely available and its innovation is to quantitatively compare the output of LA-ICP-MS data investigations to the result of an objective annotation of morphological features.

The features of FishImager will be demonstrated on the important alternative test system to animal testing, the ZFE. The recently published FishInspector software allows the automatic identification of morphological features [25]. Using this output, FishImager combines it with LA-ICP-MS results. In this study, we explore the distribution of natural elements and two xenobiotics in the ZFE. These examples point out how ablation reproducibility and toxicokinetics of xenobiotics in the ZFE can be assessed with the software. This is a step towards a more objective and reliable data analysis of LA-ICP-MS results.

## Materials and methods

### Culture of zebrafish, collection of eggs, and culture of embryos

We used the UFZ-OBI strain (*Danio rerio*, generation F13–14), obtained originally from a local breeder and kept for several generations at the UFZ. Fish were cultured and used according to German and European animal protection standards and approved by the Government of Saxony, Landesdirektion Leipzig, Germany (Aktenzeichen 75-9185.64) [26].

### Exposure experiments

Details on the example data sets, i.e., the exposures of the ZFE and the LA-ICP-MS experiments, are partly given in Halbach et al. (for the ZFE discussed in R&D section a) and the exposure to naled) [27]. In addition, ZFEs were exposed starting from 3 h post-fertilization (hpf) for 48 and 96 h to 10.3 μM 4-iodophenol (Sigma-Aldrich, CAS no. 540-38-5) dissolved in ISO-water [28]. Nine ZFEs were exposed in 18 mL of exposure medium in three replicates. pH and oxygen content of the water were measured at the beginning and end of exposure to ensure the test requirements [29]. At the end of the exposure, ZFEs were dechorionated when necessary, and five ZFEs were placed in Eppendorf tubes. Excess water was removed, and ZFEs were washed twice with 1 mL Milli-Q water. The ZFEs were carefully placed on glass slides and dried at room temperature for at least three days. Microscopic images were taken (Leica, M205 FA).

### Instrumental equipment, parameters, and data processing

Briefly, LA-ICP-MS measurements were performed with a G2 Analyte Laser (Teledyne CETAC Technologies Inc., Bozeman, MT, USA) connected to a double-focusing sector field ICP-MS based on a Mattauch-Herzog geometry (Spectro MS, Spectro, Ametek, Kleve, Germany) [30]. Daily performance and fine-tuning of the ICP-MS were performed with a NIST610 glass reference material (SRM-610, LGC Ltd., Middlesex, UK). ^12^C, ^31^P, ^39^K, ^79^Br, and ^127^I were the measured isotopes. Measurement parameters are given in Table 1. Calibration of the ^127^I signal was performed using agarose gels spiked in different concentrations with an iodide solution (Sigma-Aldrich) (see Supplementary Information (ESM) Fig. S1) [27, 31]. Merging of the transient ICP-MS signal with the position data of the laser, baseline correction, and quantification was achieved with Iolite 3.6 in Igor Pro 7.04. A table with the elemental intensities, quantified elements, and *x*,*y*-coordinates was exported as a text-file. This file (in our case around 200 KB) was imported into FishImager (termed as LA-ICP-MS data).

**Table 1:** Operational parameters of  laser ablation and ICP-MS instrumentation

ICP-MS		
RF power	[W]	1250
Cooling gas flow rate, argon	[L/min]	13.50
Auxiliary gas flow rate, argon	[L/min]	2.6
Carrier gas flow rate, helium	[L/min]	0.8
Integration time	[s]	1
Base interval	[ms]	100
Laser ablation system		
Wavelength of ArF laser	[μm]	193
Laser beam diameter	[μm]	35
Laser scan speed	[μm/s]	35
Repetition frequency	[Hz]	125
Laser fluence	[J/cm^2^]	1.77

Phenotypic features (biological ROIs) were detected with the FishInspector tool (version 1.03, Scientific Software Solutions, and Helmholtz Centre for Environmental Research) based on a microscopic image (Leica, M205 FA, denoted “reference image”) [25]. The compartment fish body (area represented by the fish contour minus the yolk) was manually added to the phenotypic features from the FishInspector software.

### Software layout

**Fig. 1:**
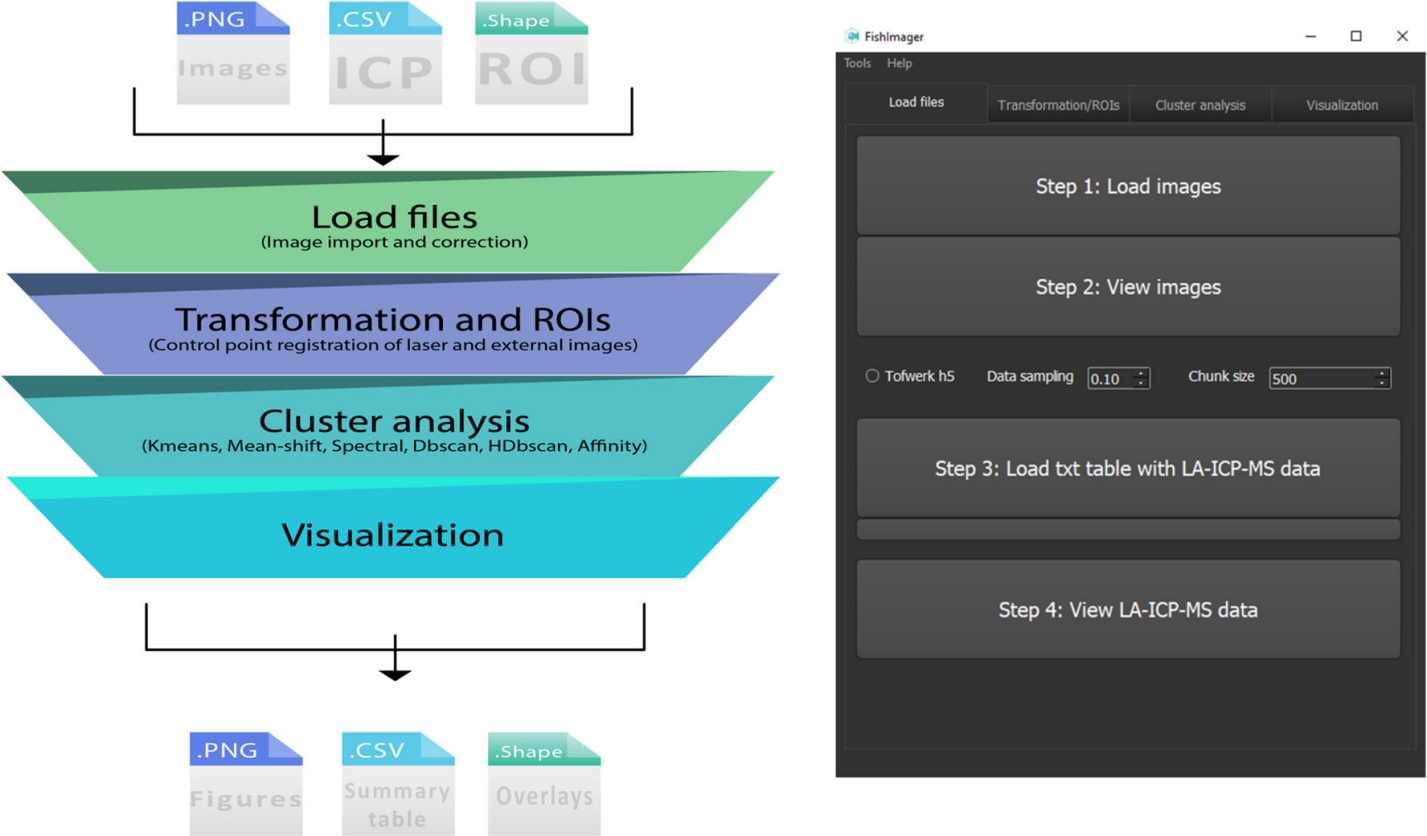
Data analysis workflow of the software FishImager (left) and exemplary layout (right)

The workflow of FishImager (Fig. 1) starts with importing the LA-ICP-MS files (camera photo, coordinate file, and LA-ICP-MS data file) and the annotated image with morphological features. The workflow also offers the option to visualize the imported data. Download information and an installation guide are available at https://git.ufz.de/holbrook/fishi-LAICPMS-Imaging-Tool. Imaging data following the structure element1, element2,…, *X* coordinate, *Y* coordinate, or the structure of an ICP-ToF-MS data file (h5-file from Tofwerk, Switzerland) can be processed. A more detailed description of the workflow is given in ESM 1. Briefly, the coordinate systems of the camera photo from the laser device and the image from FishInspector are then transformed to create an overlay of the morphological features with the LA-ICP-MS data. The morphological features assigned by the FishInspector software are automatically defined as ROIs (Fig. S2). For each ROI, quantitative information on the sum, mean, standard deviation, median, variance, minimum, and maximum can be calculated. Different cluster algorithms (k-means, mean shift, affinity propagation, spectral clustering, density-based spatial clustering of applications with noise (DBSCAN), and hierarchical dbscan (HDBSCAN), ESM Table S1) can be applied to the LA-ICP-MS data, and this cluster data can be correlated with the ROIs. The k-means clustering has been previously applied for automatic segmentation of brain sections based on elemental intensities or classification of groups of ions measured in mammalian cells using the secondary ion mass spectrometry [32, 33]. Endocrine peptides were identified in mouse pancreatic tissue using spectral clustering of matrix-assisted laser desorption/ionization-MS imaging data [34]. Details on the algorithms can be found in the description of the sklearn.cluster module for Python [35] and ESM Table S1. The information about the imaging data in the ROIs and the clustering is visualized in heatmaps. We created a graphical user interface (Fig. 1) so that the application of FishImager is not dependent on the user’s programming expertise. The input format for the assigned biological features (here the FishInspector) in FishImager may be easily edited to combine other tissue matrices with LA-ICP-MS data to develop further application areas. Extensive instruction to reproduce the data in the “Results and discussion” section is included in the ESM 2. A download link to the presented data set is also given in the git repository.

## Results and discussion

LA-ICP-MS results were explored using FishImager with respect to (a) the distribution of natural elements in the ZFE and the reproducibility between ablations of different individuals, (b) the sites of accumulation of two different xenobiotics, and (c) how uptake and distribution of one xenobiotic changes with exposure time and developmental stage. Plotted intensities of the discussed data with the scale of the ablated tissues can be found in Figs. S3, S4, and S6. Biological ROIs (fish contour, bladder, notochord, eyes, pericard, yolk) were imported from the FishInspector software (ESM Fig. S2). The ROI fish body was assigned as the difference between the ROIs fish contour and yolk. It includes the bladder, notochord, eyes, and pericard.

- Distribution of natural elements in zebrafish embryos and reproducibility between ablations of different individuals

**Fig. 2:**
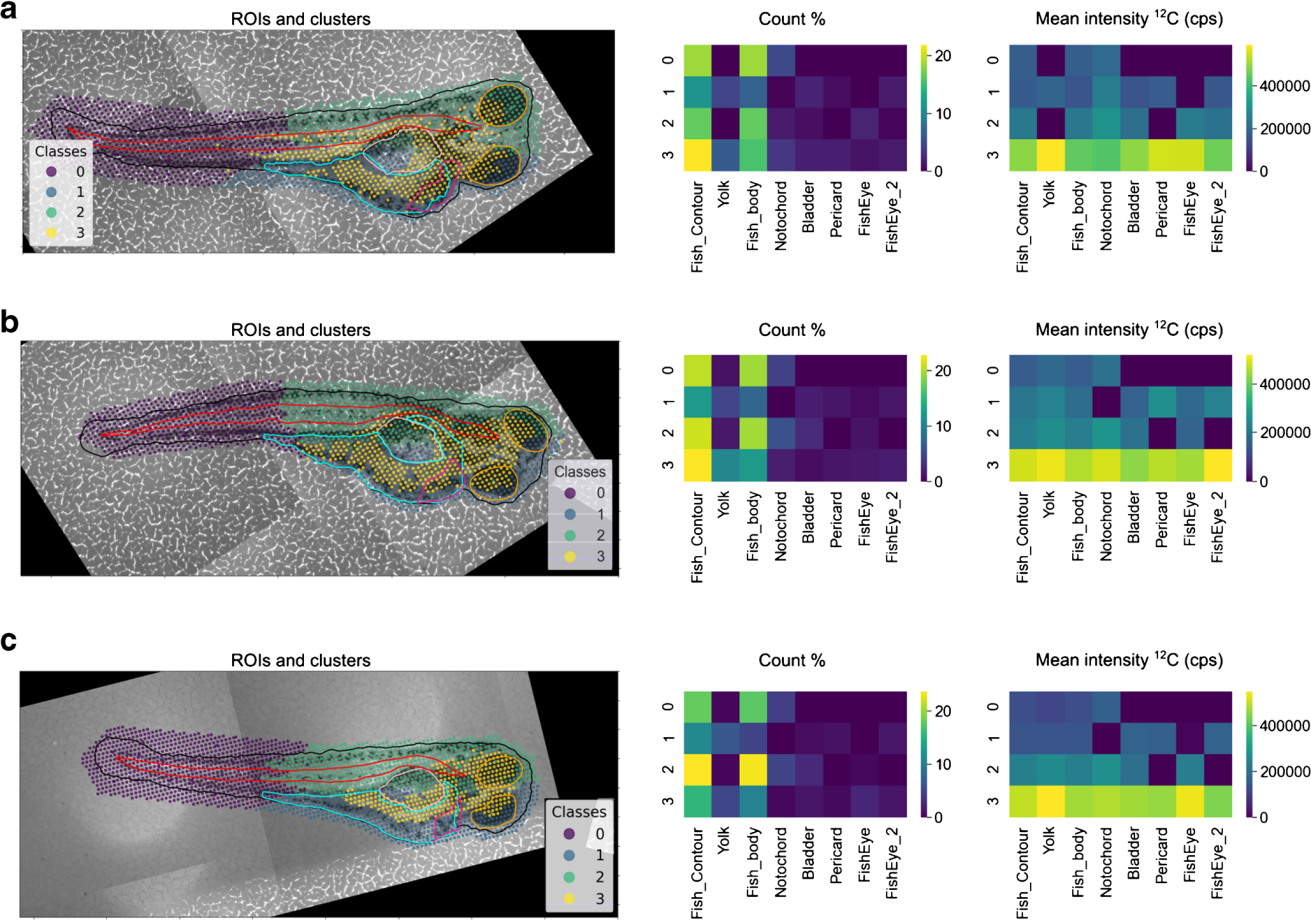
**a**–**c** K-means clustering of the ^12^C LA-ICP-MS signal intensity and the *x*- and *y*-coordinates in three zebrafish embryos. The heatmaps display the percentage of ablation area represented by the cluster and the body part (count %) and the mean intensity of the elements per cluster and per ROI (mean intensity ^12^C)

We applied FishImager to investigate the distribution of the essential elements carbon, phosphorus, and potassium in the different body compartments in ZFEs 96 hpf. These were chosen as they are accessible via ICP-MS and not present in the underlying support material. The ^12^C signal has been shown before to correlate with the height profile of the sample [27]. The clustering of the ^12^C signal between three different individual ZFEs was initially compared to gain information about the reproducibility, i.e., a combination of biological variability and measurement precision, of the three ablations (Fig. 2). The k-means algorithm was applied with the predefined numbers of clusters set to four (chosen with the elbow method [36]). Heatmaps in Fig. 2 depict the percentage of the overlapping area of the clusters and the ROIs, respectively (% counts), as well as the mean intensity in these overlapping areas (mean intensity per element). The visualization of the assigned clusters and ROIs as heatmaps is an important feature of the software, allowing easy and quick comparison between ablations and is to our knowledge not included in other available LA-ICP-MS software.

The size of cluster 3 in the ZFEs varied from 16 to 23% of the ablation area between the individuals (Table 2, Fig. 2). This cluster always contained the highest mean ^12^C intensity (heatmaps of cluster 3 for the mean intensity ^12^C in Fig. 2). The highest mean ^12^C intensity overlapped with the ROIs fish eyes and the yolk. The precision of the carbon distribution among the three replicates can be assessed, e.g., by performing correlation analysis among the heatmaps of the replicates. These contain the information on the clustering and the ROI assignment. The three heatmaps of the mean ^12^C intensity showed a significant correlation (Spearman’s correlation coefficients between the individuals 0.71, 0.77, 0.91, *p* < 0.001 for all coefficients); e.g., the mean ^12^C intensities in cluster 3 and the ROI fish body were 4.47 ± 0.94, 4.61 ± 0.73, and 4.74 ± 0.98 × 10^5^ cps. Therefore, we conclude that the percentage of the overlap of the clusters and the ROIs, thus the spatial distribution of the ^12^C signal, showed a good agreement between the different replicates. The highest difference between the three individuals was observed for ^12^C in cluster 2 and the ROI yolk in individual 1. This can be explained by the different number of pixels associated with cluster 2 and the ROI yolk (Fig. 2, middle column). Cluster 2 of individual 1 does not overlap with the ROI yolk. In contrast, very few LA-ICP-MS pixels are classified as cluster 2 and ROI yolk for individuals 2 and 3 resulting in the difference between individuals.

**Table 2:** Cluster sizes as a percentage of total ablation area and mean signal intensities (cps) for the elements ^12^C, ^39^K, and ^31^P for three single embryos (*n* = 1, individual 1 to 3)

	Individual 1						Individual 2		Individual 3	
Cluster	^12^C		^39^K		^31^P		^12^C		^12^C	
	Percentage of ablation area (%)	Mean signal intensity (cps)	Percentage of ablation area (%)	Mean signal intensity (cps)	Percentage of ablation area (%)	Mean signal intensity (cps)	Percentage of ablation area (%)	Mean signal intensity (cps)	Percentage of ablation area (%)	Mean signal intensity (cps)
0	36.8	1.31 × 10^5^	32.2	6.17 × 10^5^	23.9	1.26 × 10^5^	31.5	1.33 × 10^5^	37.2	1.03 × 10^5^
1	18.3	1.36 × 10^5^	24.0	2.73 × 10^6^	26.7	1.37 × 10^5^	16.6	1.88 × 10^5^	20.0	1.23 × 10^5^
2	22.9	1.96 × 10^5^	18.2	3.36 × 10^5^	23.2	1.32 × 10^4^	29.4	1.84 × 10^5^	27.3	2.18 × 10^5^
3	22.0	4.88 × 10^5^	25.6	1.37 × 10^6^	26.2	5.47 × 10^4^	22.5	4.81 × 10^5^	15.5	4.99 × 10^5^

**Fig. 3:**
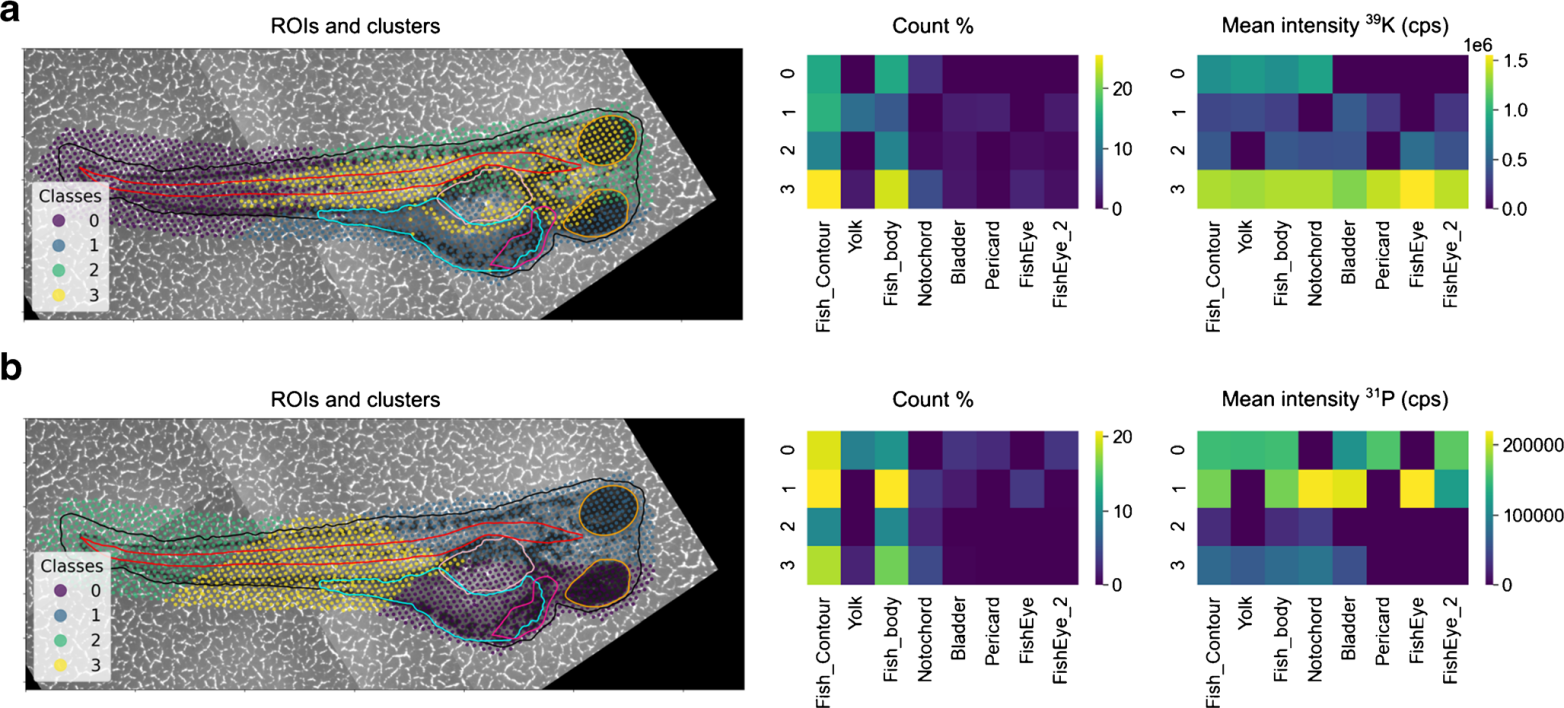
Assignment of four clusters by the k-means algorithm of the *x*- and *y*-coordinates and the LA-ICP-MS intensity data for (**a**) ^39^K and (**b**) ^31^P, respectively, for a 96 hpf zebrafish embryo. The LA-ICP-MS data are color-coded into the four clusters (first column). Outlines of the biological ROIs are displayed. The heatmaps display the percentage of the ablation area represented by the cluster and the biological ROIs (count %) and the mean intensity of the elements per cluster and per ROI (mean intensity ^39^K/^31^P)

By using k-means clustering, the elements ^31^P and ^39^K were also assigned to four clusters, respectively (Fig. 3). The element ^39^K partitioned into clusters 0 to 3; three of them (0 to 2) were evenly distributed over the ablation area and consisted of 18 to 32% of the total ablation area (Table 2). The fourth cluster (cluster 3, 26% of the ablation area) contained the highest mean intensity of the element per cluster (Table 2). The highest mean intensity of ^39^K overlapped with the ROI fish eyes (Fig. 3a). A comparison of the ^12^C and ^39^K distribution showed a significant correlation (Spearman’s correlation coefficients 0.77, *p* < 0.001) in various ROIs except for the ROI yolk. While the ROI yolk is mainly represented by cluster 3 for ^12^C with the highest mean intensity, it is represented by cluster 1 for ^39^K with a comparable low mean intensity (Figs. 2a and 3a). This confirms that the ^12^C signal may be used to reflect best the varying tissue densities of the ablated material as the yolk is a lipid- and protein-rich body compartment [27, 37]. The potassium channels are expressed mainly in the brain and trunk, which could be why the different distribution patterns of ^39^K and ^12^C were observed [38]. The element ^31^P was partitioned into four clusters of nearly equal size, which were evenly distributed over the ZFE (Table 2, Fig. 3b). The ^31^P signal intensity did not distribute in a specific body compartment. This is illustrated by the heatmap showing that no cluster overlaps with all the body compartments as for ^39^K and ^12^C; e.g., the ROI fish eyes are not represented by the same cluster as before for ^12^C and ^39^K.

The examples of the spatial distribution of the natural elements in the ZFE impressively illustrated the possibility of FishImager to calculate and visualize the means of the elemental distribution in several individual ZFEs. This forms the basis for routinely applying metrology principles (such as reproducibility) and performing statistical tests, e.g., to test for the significance of differences between different groups, for example, exposed and non-exposed organisms, compared to the difference between individuals within one group.

(b) Distribution of xenobiotics in the zebrafish embryo

FishImager allows analyzing correlations in the spatial distribution of different elements in one organism. Identifying positive or negative correlations of elements may be used to link exogeneous with endogenous elements, e.g., platinum-based anti-cancer compounds or tattoo ink [7, 39]. For demonstration, the distribution pattern and accumulation of two xenobiotics in the ZFE are explored. The two compounds contain characteristic elements suited for their determination by LA-ICP-MS: the bromine-containing compound naled and 4-iodophenol. Naled was shown to be an acetylcholinesterase inhibitor, and 4-iodophenol is presumably a baseline toxic compound that accumulates in the yolk of the ZFE [27, 40]. By applying the k-means algorithm, the baseline-corrected bromine or iodine intensities together with the carbon signal (as a thickness measure since whole ZFEs are ablated [27]) and the spatial information were partitioned into 5 clusters for both exposures, respectively (selected based on the elbow method).

**Fig. 4:**
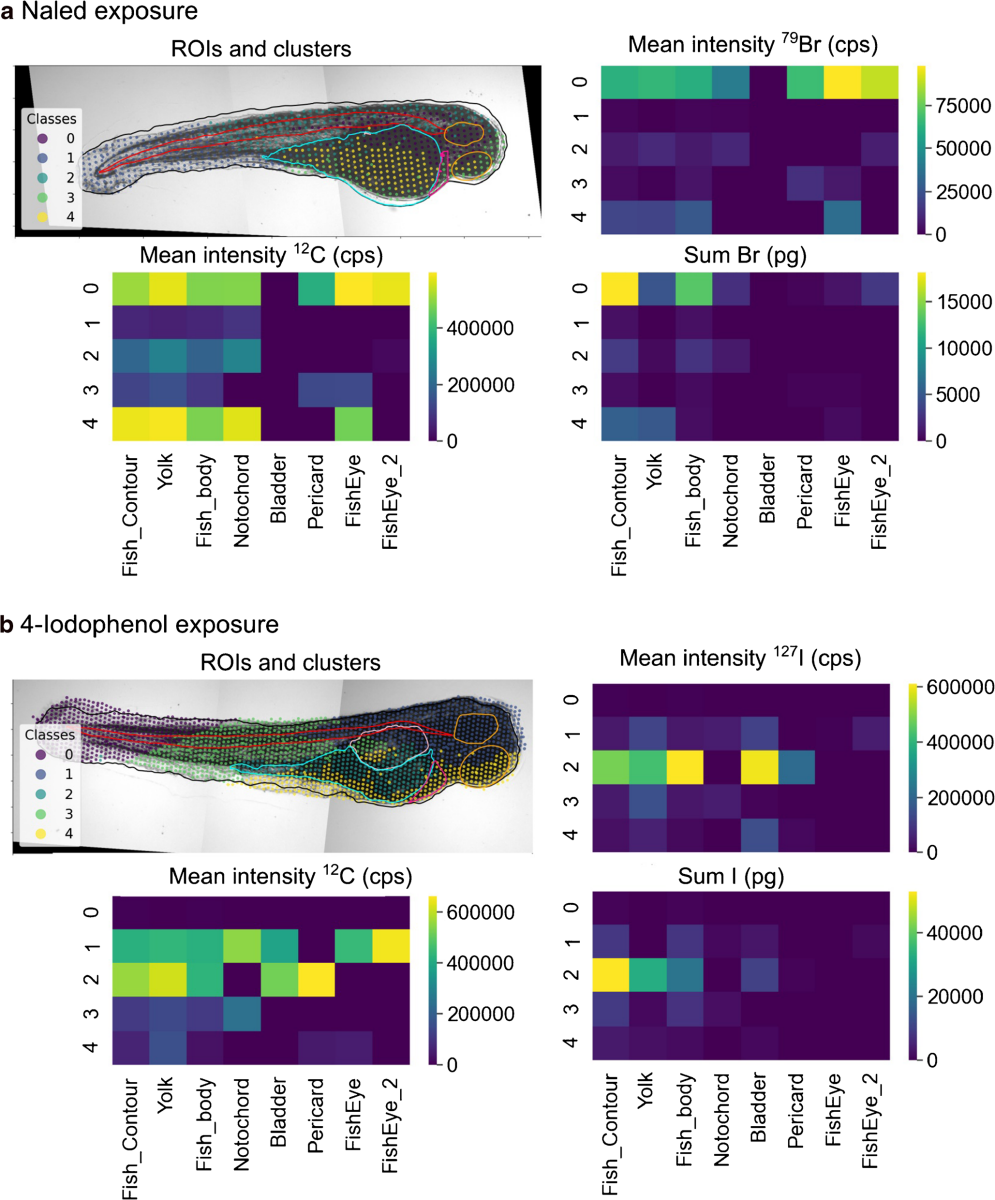
K-means clustering of (**a**) the bromine and carbon intensities and the *x*,*y*-coordinates for the 24 h exposures with the bromine-containing naled (72 to 96 hpf) and (**b**) the iodine and carbon intensities and the *x*,*y*-coordinates for the 96-h exposure (0–96 hpf) with 4-iodophenol. The heatmaps display the mean intensity of each elements per cluster and per ROI (mean intensity ^79^Br/^12^C/^127^I) and the quantified bromine and iodine amount per cluster and ROI (sum Br, I)

In the naled exposure, clusters 1 and 3 had the lowest mean bromine intensity (1.37 × 10^3^ cps and 2.73 × 10^3^ cps, Fig. 4) and together summed up to 42% of the area (Table 3). The tissue density was also lowest in these two clusters and represented the tail of the ZFE and partly one eye. Cluster 2 with medium bromine and carbon intensity overlapped with the ROI fish body and partly included the notochord. Interestingly, cluster 4 (15% of the ablation area) with the highest tissue density (mean intensity ^12^C: 5.82 × 10^5^ cps) overlapped mainly with the ROI yolk (ESM Fig. S5a) but did not contain the highest mean bromine intensity. The highest mean bromine intensity was assigned to cluster 0 (6.30 × 10^4^ cps) and represented the ROI head of the ZFE body, including the ROIs notochord and fish eyes.

**Table 3:** Cluster sizes as a percentage of total ablation area for the naled and the 4-iodophenol exposure of zebrafish embryos (k-means clustering). The mean LA-ICP-MS intensities of ^79^Br, ^127^I, and ^12^C in the clusters and the quantified bromine and iodine amounts are displayed

Cluster			Naled exposure			4-Iodophenol exposure		
	Percentage of ablation area (%)	Mean intensity ^79^Br (cps)	Mean intensity ^12^C (cps)	Total Br (ng)	Percentage of ablation area (%)	Mean intensity ^127^I (cps)	Mean intensity ^12^C (cps)	Total I (ng)
0	17.7	6.30 × 10^4^	5.09 × 10^5^	18.2	21.4	2.39 × 10^3^	4.29 × 10^3^	0.48
1	29.8	1.37 × 10^3^	6.33 × 10^4^	0.88	25.8	3.56 × 10^4^	3.80 × 10^5^	8.51
2	25.3	7.22 × 10^3^	1.99 × 10^5^	2.90	11.3	4.86 × 10^5^	5.60 × 10^5^	53.1
3	12.1	2.73 × 10^3^	1.12 × 10^5^	0.70	23.7	4.00 × 10^4^	1.06 × 10^5^	8.78
4	15.1	2.07 × 10^4^	5.82 × 10^5^	5.60	17.8	2.25 × 10^4^	5.70 × 10^4^	3.74

Provided that the LA-ICP-MS system is adequately calibrated [27, 31], the amount of an analyte in the respective ROI can be quantified (Table 3 and Fig. 4). The bromine amount in the ROI fish body is mainly accumulated in the notochord and the fish eyes. It was assumed that this accumulation originates from the neuroactive toxicity of naled [27]. By applying FishImager, the previously published [27] manual ROI assignment could now be automatically achieved, and single body parts were linked to the bromine accumulation. Previous results are confirmed and could be specified here with the localization of the bromine in the notochord and fish eyes.

For the ZFEs exposed to 4-iodophenol, the iodine intensity distribution and the LA coordinates partitioned into 5 clusters: clusters 0, 1, 3, and 4 represented each between 18 and 26% of the total pixels; cluster 2 only 11% (Table 3). The latter cluster contained the highest mean iodine intensity (4.86 × 10^5^ cps), which was one magnitude higher than in the other clusters. This cluster is situated between the ROIs yolk and body. Seventy percent of the total iodine amount accumulated in cluster 2. It is suggested that the gastrointestinal (GI) tract develops in this region. A localization of iodine in the GI tract may be linked to biotransformation of 4-iodophenol to other iodine-containing transformation products or the elimination via the GI tract.

Localizing the site of enrichment of a toxicant (or its metabolites) within an organism and quantifying its amount in different organs is needed for toxicokinetic and toxicodynamic studies. The incorporation of morphological ROIs is an advantage of FishImager compared to available software. It may improve the understanding of toxicity mechanisms (e.g., by enrichment of a neurotoxic compound in the brain and notochord) and outline that a toxicant is metabolized (e.g., by enrichment in the liver) or eliminated via the GI tract.

**Fig. 5:**
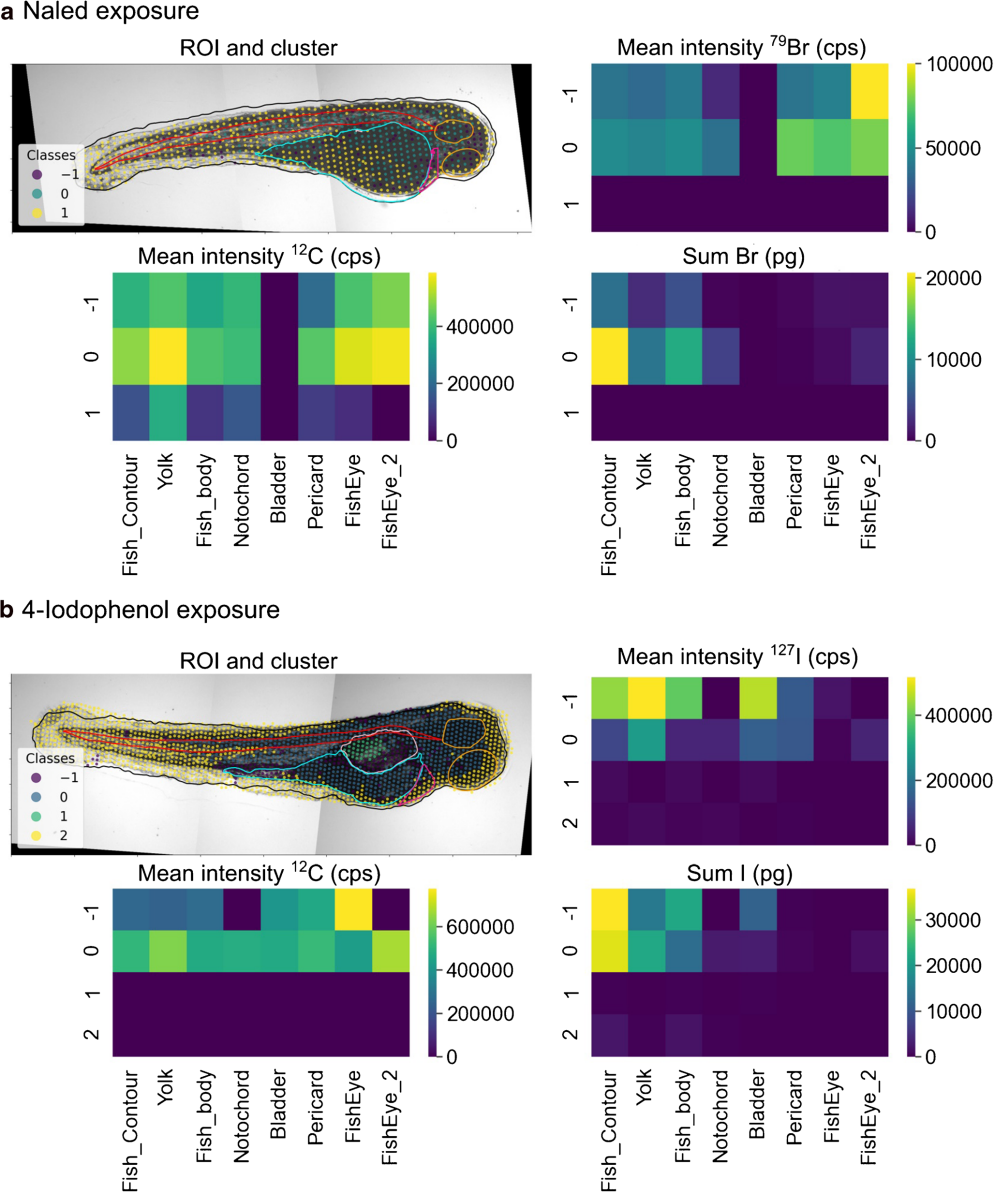
HDBSCAN clustering of (**a**) the bromine and carbon intensities and the *x*,*y*-coordinates for the 96 h exposure to the bromine-containing naled (minimum cluster size set to 7) and (**b**) the iodine and carbon intensities and the *x*,*y*-coordinates for the 96 h exposure to 4-iodophenol (minimum cluster size set to 22). The heatmaps display the mean intensity of each element per cluster and per ROI (mean intensity ^79^Br,^12^C,^127^I) and the total amount in the segments (sum)

In these two examples, the k-means algorithm was applied for the cluster analysis; it was previously used, e.g., to reveal different elemental fingerprints in brain tissue [32]. For statistical analysis, three partitioning (k-means, affinity propagation, spectral clustering) and three cluster (mean shift, DBSCAN, HDBSCAN) algorithms are provided by the software. These algorithms have different data knowledge requirements as well as different capabilities in coping with noisy data. An overview of their features is given in ESM Table S1. Furthermore, specific cluster parameters depending on the algorithms can be controlled. When applying cluster algorithms other than k-means, such as the HDBSCAN, to the data from the two examples above, the resulted segmentation differed (Fig. 5). In the case of the naled exposure, three clusters were obtained by the HDBSCAN. The cluster − 1 with a mean bromine intensity of 3.82 × 10^4^ cps contained 12% of the pixel and may represent one fish eye and possibly measurement outliers (Fig. 5a; ESM Fig. S5 and Table S2). The ROIs eyes, notochord, pericard, and partly the yolk overlapped with cluster 0 of a similar mean bromine intensity (4.76 × 10^4^ cps, 26% of the ablation area) in contrast to cluster 1 representing the rest of the ROI yolk and tail of the ZFE with a mean bromine intensity of 0 cps.

For the ZFE exposed to 4-iodophenol, the iodine and carbon intensity and the coordinates were assigned to four clusters based on HDBSCAN. Cluster − 1 displayed the GI tract with the highest mean iodine intensities of 4.20 × 10^5^ cps (in total 37 ng, 9% of the ablation area, ESM Table S2). Cluster 0 had the second-highest mean iodine intensity (1.08 × 10^5^ cps, 35.2 ng, 34% of the ablation area, ESM Table S2) representing the ROIs yolk and partly the fish body. Cluster 1 only included 2% of the ablation area and embodied the ROI bladder (mean iodine intensity 1.46 × 10^4^ cps). Cluster 2 represented the rest of the ZFE with a low iodine intensity (mean 4.33 × 10^3^ cps).

The HDBSCAN algorithm resulted in better differentiation between the body compartments of the ZFE with high iodine or bromine intensities and those compartments containing very low or no signal intensity than the k-means clustering. This conclusion can be made as knowledge on the anatomy of the biological tissue existed. FishImager offers the flexibility to test different cluster algorithms, and it may be useful to test these on the same data to gain more robustness of the clustering result.

(c) Identification of changes over time in the distribution of a xenobiotic in the zebrafish embryo

**Fig. 6:**
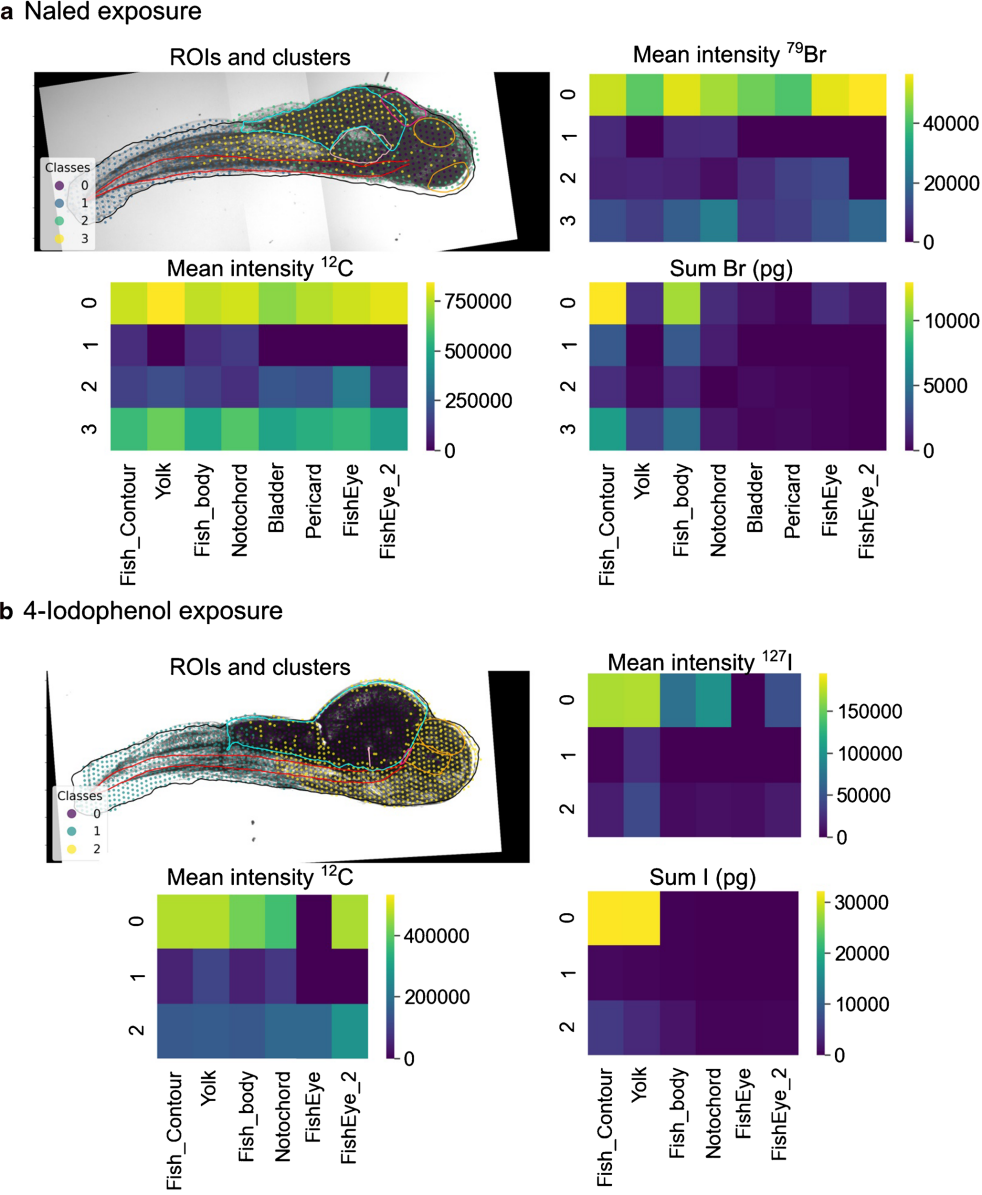
K-means clustering of the (**a**) bromine and carbon intensities and the *x*,*y*-coordinates for a 4 h exposure (92 to 96 hpf) to naled (cluster number set to 4) and (**b**) iodine and carbon intensities and the *x*,*y*-coordinates for the 48 h exposure (0 to 48 hpf) to 4-iodophenol (cluster number set to 3). The bladder and pericard are not displayed for (**b**) because for this developmental stage, it is not meaningful. The heatmaps display the mean intensity of the elements per cluster and per ROI (mean intensity ^79^Br, ^127^I, ^12^C) and the quantified amount per cluster and ROI (sum Br, I)

In addition to the identification of accumulation patterns and correlation of elements, toxicokinetics, i.e., determination of time-resolved changes in internal amounts and distribution, can be supported by FishImager. The distribution of the bromine-containing compound naled from section (b) for the 24 h exposure is compared to a shorter exposure time (4 h, Fig. 6a). The k-means clustering resulted in four clusters (Fig. 6a). Cluster 0 represented the highest mean bromine and carbon intensity (5.34 × 10^4^ cps and 7.79 × 10^5^ cps, ESM Table S3) and overlapped strongest with the ROIs fish eyes and notochord in the fish body (ESM Fig. S7). The total quantified bromine in cluster 0 was 13 ng after 4 h (ESM Table S3) and increased only slightly to 18 ng in the same body compartments after 24 h of exposure (Table 3, cluster 0). Thus, neither the site of the accumulation nor the target site of the toxicity appears to change with exposure duration.

The k-means clustering was also applied with three chosen clusters (confirmed with the elbow method) for a shorter exposure period of 4-iodophenol in a younger ZFE (48 h, 48 hpf, Fig. 6b). Cluster 1 and cluster 2 mainly represented the ROI fish body of the ZFE with the ROIs notochord and eyes (ESM Fig. S7). The ROI yolk was primarily represented by cluster 0. The mean iodine and carbon intensity are also highest in this cluster (ESM Table S3). At shorter exposure times, 32 ng iodine accumulated in the ROI yolk (cluster 0) increasing to 53 and 4 ng in the GI tract and ROI yolk at longer exposure times (see Table 3 and ESM Table S2). Contrary to the bromine case, the 4-iodophenol showed a more dynamic behavior: With continuing exposure and ZFE development, the iodine is transferred from the yolk to the GI tract. This may be explained by an increased metabolizing capacity of older ZFEs [42, 43] as well as the development of the elimination pathways via the liver and kidney [44]. It has recently been shown that the distribution of toxicants between the yolk and the ZFE body of developing ZFEs can be highly dynamic [40]. Imaging mass spectrometry in combination with FishImager allows direct access to the amount of the toxicant in the yolk.

## Conclusions

The developed workflow with its implementation in the software FishImager combines quantitative LA-ICP-MS imaging data with annotated biological features. This was demonstrated here for the distribution of natural elements as well as for two xenobiotics in the model organism ZFE with its morphological features obtained by the FishInspector software. Namely, (i) the reproducibility and biological variation of LA-ICP-MS results were assessed by calculating localized mean intensities of the elements and performing a correlation analysis among replicates; (ii) different cluster algorithms were applied on the LA-ICP-MS data and visualized; (iii) biological ROIs were imported, automatically allocated to the LA-ICP-MS data and combined with the cluster results. This combination makes it possible to move from visual observations and interpretations towards validation and statistical proof and an automatic, more reliable ROI definition. Results can be compared in heatmaps. Finally, the data evaluation time can be significantly reduced using FishImager.

The built-in tool for importing and processing LA-ICP-ToF-MS data increases the application possibilities of these simultaneous multielement detection techniques. The developed tool can be applied in the future to other solid or soft matrix samples and data from different imaging methods. For example, brain sections [45] annotated with morphological features may be combined with MS imaging data in this freely available software. Moreover, the approach may also be applied to other important test organisms such as *Daphnia magna* or *Xenopus* which are used for instance in toxicokinetic studies and risk assessment of chemicals [41, 46] but also for quality control in material development where the distribution of elements is crucial, for instance in composite materials. Ultimately, the developed workflow allows a combination of different imaging data and its systematic and reproducible interpretation.

## Supplementary information

Supplementary information 1. (Supplementary_information_1.docx) Additional information on the LA-ICP-MS calibration and FishImager results of the examples presented in the manuscript. (PDF 1469 kb)

Supplementary information 2. (Supplementary_information_2.pdf) Tutorial for using FishImager and reproducing the examples in the manuscript. (PDF 1335 kb)

## Data Availability

The data sets supporting the conclusions of this article are included within the additional files of this article or linked to the project homepage.
