# Supplementary material for: Effective processing and evaluation of chemical imaging data with respect to morphological features of the zebrafish embryo
Source: Anal Bioanal Chem. 2021 Feb 1;413(6):1675–87. doi: 10.1007/s00216-020-03131-4 (PMC7921040; doi:10.1007/s00216-020-03131-4)
Supplement: Supplementary file 1 — (Supplementary_information_1.docx) Additional information on the LA-ICP-MS calibration and FishImager results of the examples presented in the manuscript. (PDF 1469 kb) [file 216_2020_3131_MOESM1_ESM.pdf]

Supplementary information

Effective processing and evaluation of chemical imaging data with respect to morphological features of the zebrafish embryo

Halbach, K., Holbrook, T., Reemtsma, T., Wagner, S.

Department of Analytical Chemistry, Helmholtz Centre for Environmental Research - UFZ, 04318 Leipzig, Germany

Institute of Analytical Chemistry, University of Leipzig, 04103 Leipzig, Germany

*Corresponding author: Stephan Wagner

E-mail address: Stephan.wagner@ufz.de

Calibration of LA-ICP-MS data

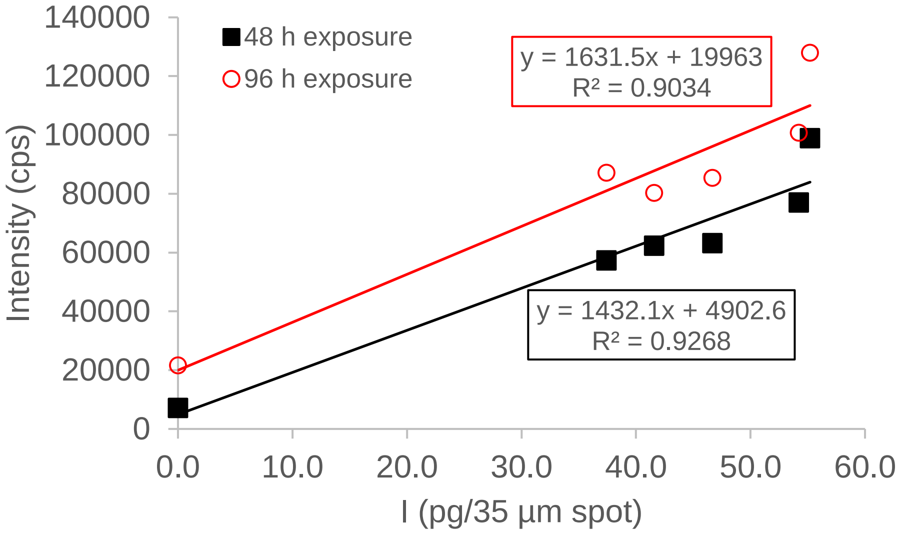

Fig. S1 Calibration curves with a spotsize of 35 µm conducted on the day of the ablations of the embryos exposed from 2-50 hpf (black square) and 2-98 hpf (red dot). The unit of the x-axis refers to the iodine content of the agarose standards and the corresponding intensity is displayed on the y-axis. Limit of detection = 14.2 x 10^3 cps (black) and 29.8 x 10^3 cps (red); limit of quantification = 30.5 x 10^3 (red) and 48.92 x 10^3 (black)

Implementation in FishImager

Load files

The image taken by the camera of the LA-system (Analyte G2, Teledyne CETAC Technologies) (termed "laser camera image") and the corresponding file with the coordinates and image size are imported (*Step 1: Load images*). The two files have the format produced by Chromium 2.4 (Teledyne Photon Machines). Additionally, the image used to create the morphological features (termed "reference image") is imported. Both imported images can be viewed in *Step 2: View images*. The transient ICP-MS with the laser coordinates are imported as a tab-delimited text file in *Step3: Load txt table with LA-ICP-MS data*. The text file is structured with columns (element1, element2, …, X coordinate, Y coordinate) and rows containing the measured data (see link to cloud with example files in https://git.ufz.de/holbrook/fishi-LAICPMS-Imaging-Tool/-/tree/master/test_data). Currently, the file format of Tofwerk (.h5) is also supported. For other file formats, either these need to be transformed into the same structure or programming adjustments in FishImager are needed.

We have also included the option to load ICP-ToF-MS data as an H5-file. This is the output file of TOFpilot (version 2.5.4.1) and TofDaq (Tofwerk, Switzerland) with all selected m/z values from the measurement. As the large file size requires a high computing time, data sampling is implemented as a float value to downsample the buff/write coordinates. Due to the limitation of the large list iterations, chunk size allows the user to select a size of data chunk for increased iterations performance. The file size tested had a dimension of 1144 x 50 x 1 x 315; larger dimensions were not tested. An exemplary workflow for one h5-file is shown in ESI 2.

The imported LA-ICP-MS data can then be checked with *Step 4: View LA-ICP-MS data*. The channel which is displayed can be selected in the drop-down menu. To view another channel, close the figure and select the new one in the drop-down menu, and press *Step 4* again.

Transformation and ROI

The aim of this tab is first to transform coordinates of the LA-ICP-MS data to fit the reference image (or another image of the same sample) from which the ROIs are determined. This allows combining two independent analyses of the biological specimen (here LA-ICP-MS and the FishInspector tool). Secondly, the LA-ICP-MS data are combined with the data of the morphological features.

The registration of the two images is performed by choosing first a point in the laser camera image, then the corresponding one in the reference image, and then repeating this for a minimum of 3 points (*Step 1: Choose control points*). The algorithm produces an affine transformation matrix of the two images for registration of the laser camera image (source, src) to the reference image (destination, dst). Different registration methods are available by changing the parameter in the code from affine to the desired algorithm (https://scikit-image.org/docs/dev/api/skimage.transform.html#skimage.transform.estimate_transform). The overlay is visible in *Step 2: Image registration*. The same transformation is performed with the coordinates of the ablation pattern in the text file with the LA-ICP-MS data.

The data of morphological features (here the SHAPES.JSON file from the FishInspector) are imported in *Step 3: Load JSON file and create ROIs*. This file format is the _Shapes.JSON from the FishInspector software and contains the outline coordinates of the different assigned body parts.[1] This module of the FishImager software may be adjusted to different referencing methods for other applications.[2] The imported ROIs are plotted over the ablated area of the LA-ICP-MS data in *Step 4: Display LA-ICP-MS data together with ROIs*.

Cluster analysis

After these steps, the data are ready for further exploration. From the imported LA-ICP-MS data table, you can select the elements for clustering, which are then normalized using a min_max_Scaler. You may also include the x- and y-coordinates as variables. This is an advantage of FishImager as not only the identification of similar elements distribution but also homogenous areas of single elements is possible.

Six different cluster algorithms have been implemented: k-means, mean shift, affinity propagation, spectral clustering, DBSCAN, and HDBSCAN can be chosen depending on the purpose and preknowledge of the data (Table S1). The cluster parameters can be chosen and varied in FishImager, e.g., number of clusters for k-means, spectral clustering, and HDBSCAN, epsilon for DBSCAN. For the k-means clustering, we have also implemented a plot showing the sum of squared errors to determine the optimal number of clusters (elbow method[3]).

Table S1 Overview of the available cluster algorithms in FishImager and adjustable parameters.

Cluster algorithm	Description	Parameters to be known	Application
K-means	Algorithm that partitions your data in a predefined number of groups with equal variance (within-cluster sum-of-squares), all data points are grouped incl. noise	number of clusters	General purpose, also for large data sets applicable, even cluster size
Mean shift	Centroid based method, returns clusters and not partitions/groups, does not cluster every point, slow algorithm	bandwidth	Many clusters with uneven cluster size
Affinity propagation	Data votes for their preferred exemplar, graph distance, problematic for noisy data (similar to k-means), slow algorithm, so not recommended for large datasets	damping, sample preference	Many clusters, uneven cluster size, non-flat geometry
Spectral clustering	Graph clustering based on graph distance (e.g., nearest-neighbor graph), partitioning algorithm, includes noise	number of clusters	Small number of clusters, even cluster size
DBSCAN	Density based algorithm, extracts dense clusters and excludes background data as noise, problems with clusters of varying density.	Epsilon as a distance value	Large data sets, uneven cluster size
HDBSCAN	Allows varying density clusters, other similar to DBSCAN, high stability	Minimum cluster size	Large data sets, uneven cluster size

Visualization

The obtained clusters and the assigned ROIs are visually overlaid in one figure (*Step 1: Visualization of the clusters and the ROIs*). Next, a table (*Step 2: Calculate table with descriptive statistics*) with the descriptive statistics of a chosen elemental channel for the different clusters is compiled: sum, mean, standard deviation, median, variance, minimum and maximum. The total pixel number and percentage of the ablation area in the clusters are shown in a table using the *Cluster counts* function.

Heatmaps then visually merge the cluster and ROI data. The heatmap *Total ion count (sum)* may be used to look at the sum of the selected elemental channel per cluster and ROI. This heatmap contains absolute values; the percentage can be viewed in *%Total ion count per cluster*. Important information may not only be the sum of the selected channel but also the mean. The heatmaps *Total ion count (mean)* and *Total ion count (std)* show the color-coded mean and standard deviation of the elemental channel per cluster and per ROI, respectively. Another information shown is the number of counts of the LA-ICP-MS data per cluster normalized to the shape of the data matrix as a percentage (*Count %*). The underlying data for the heatmaps can be exported by using the *Export CSV* function. Additionally, a table with the percentage areas of the ROIs can be compiled (*ROI areas*).

Annotation of morphological features of the zebrafish embryo

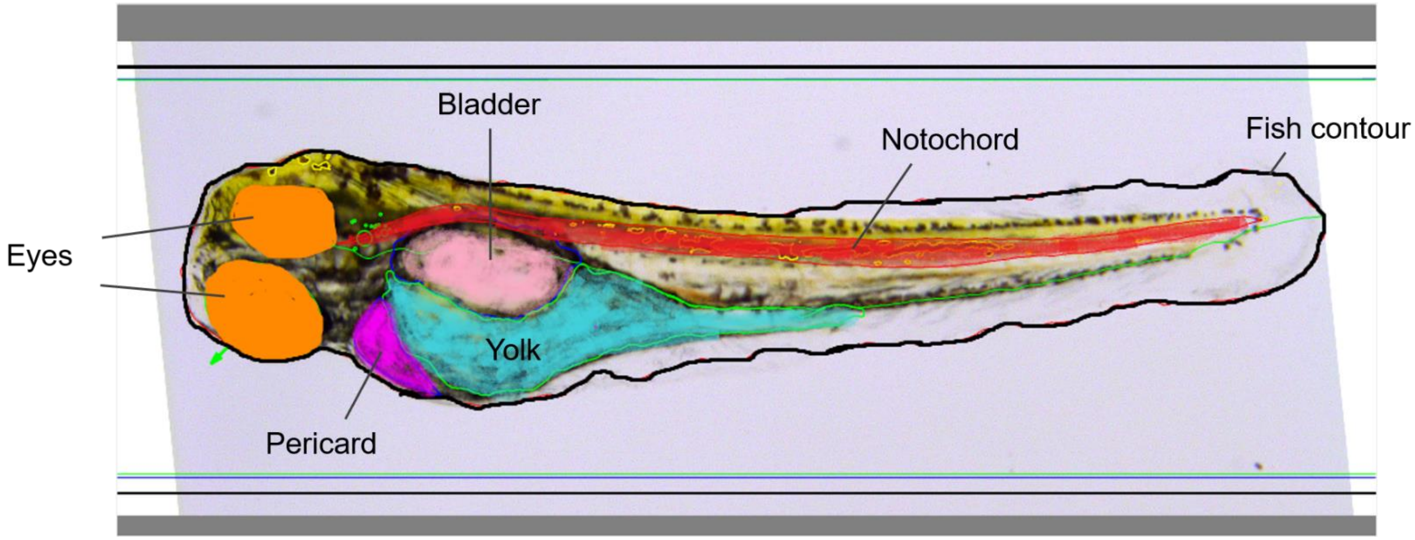

Fig. S2 Annotated zebrafish embryo by the FishInspector software. The embryo displayed is 96 hours post fertilization. In the manuscript, the term “fish body” refers to the area inside the fish contour minus the yolk.

Distribution of natural elements in zebrafish embryos and reproducibility between ablations of different individuals

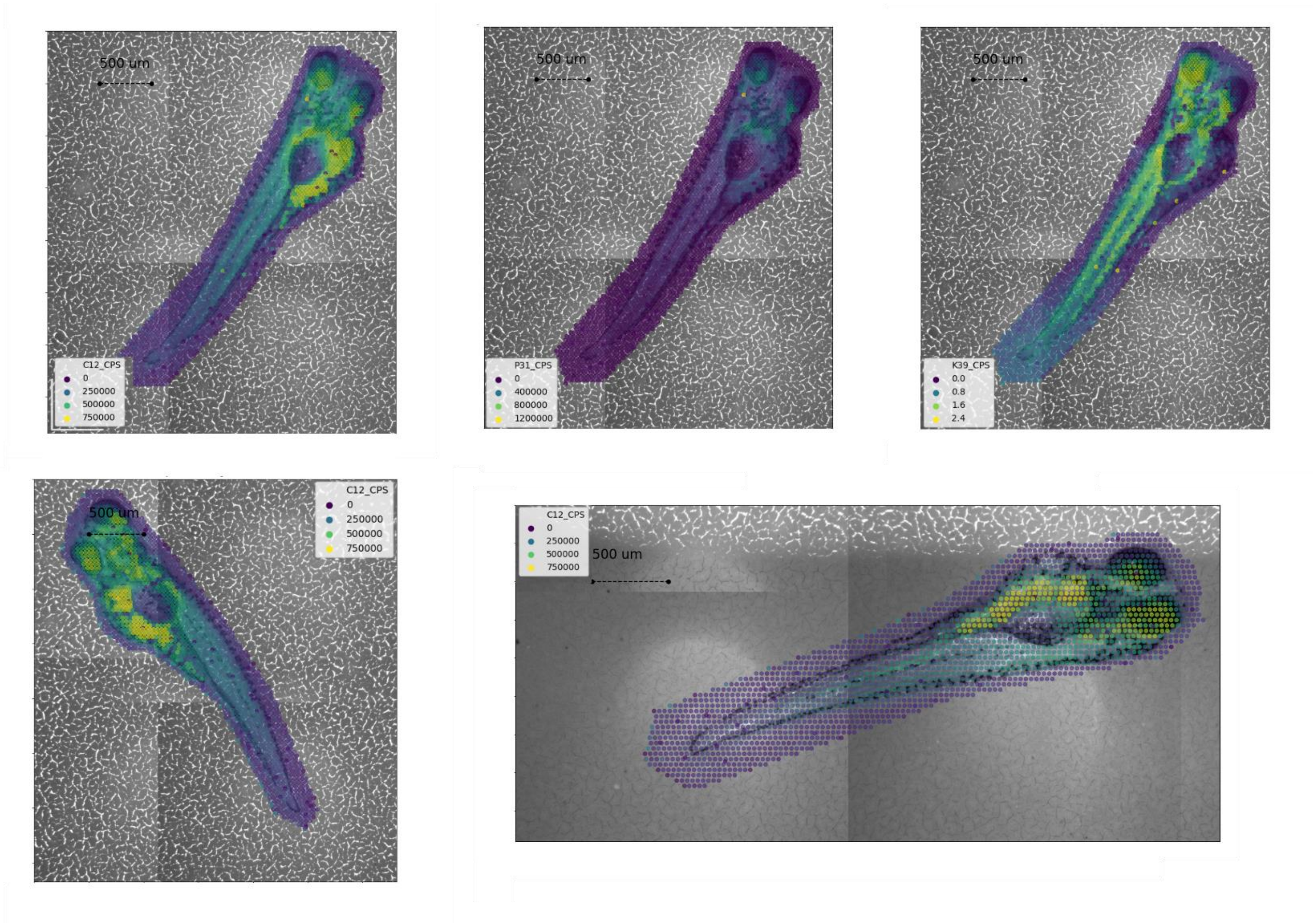

Fig. S3 Intensities of the baseline-corrected carbon (^{12}C), phosphorus (^{31}P), and potassium (^{39}K) LA-ICP-MS signals for zebrafish embryo individuum 1 (first row). Intensities of the baseline-corrected carbon (^{12}C) for zebrafish embryo individuum 2 (second row left) and 3 (second row right). Scale bars indicate 500 μm.

Distribution of xenobiotics in the zebrafish embryo

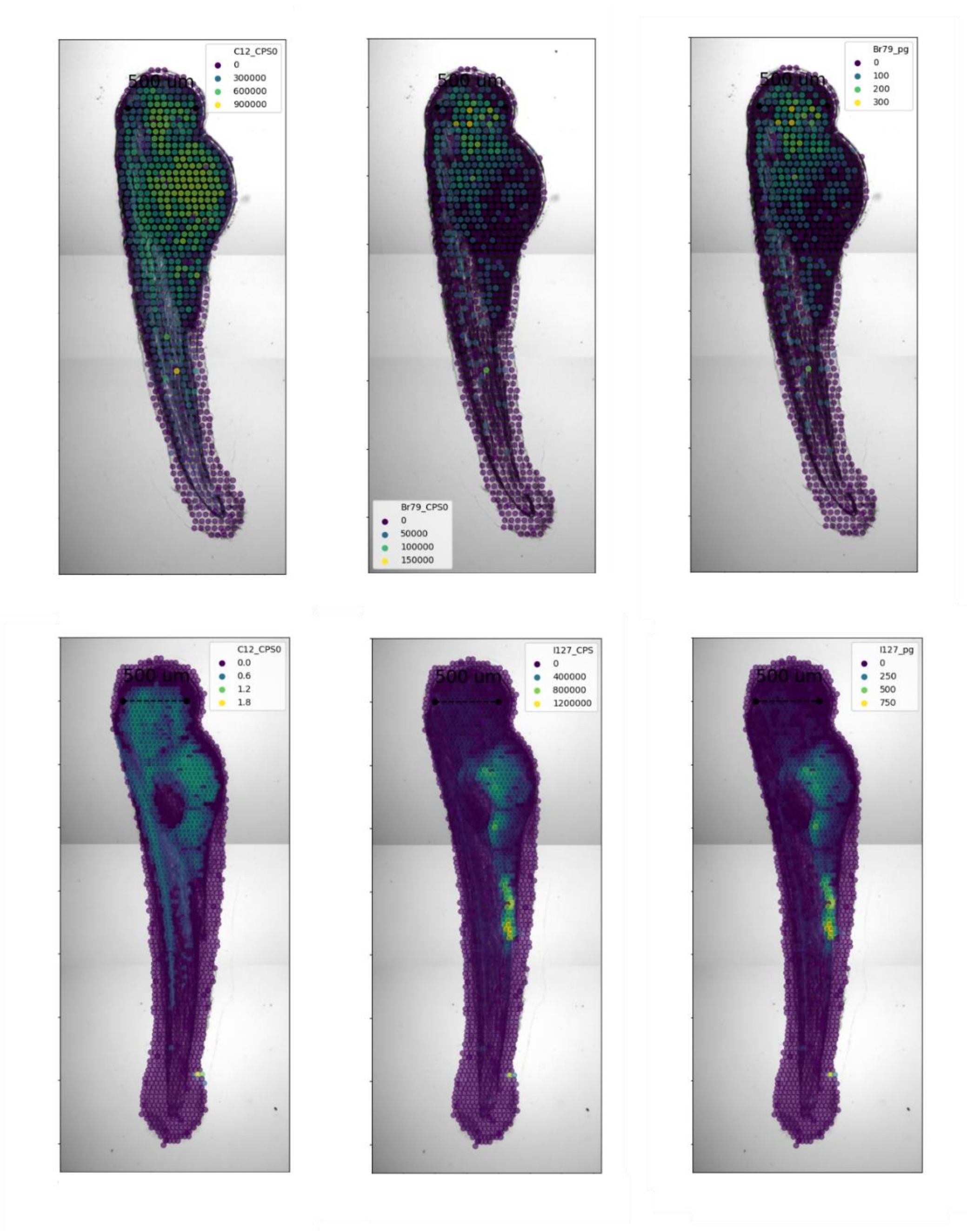

Fig. S4 Intensities of the baseline-corrected carbon (^{12}C), bromine (^{79}Br), and quantified bromine (in pg) LA-ICP-MS signals for the 24 of the zebrafish embryo (first row). Intensities of the baseline-corrected carbon (^{12}C), iodine (^{127}I), and quantified iodine (in pg) LA-ICP-MS signals for the 96 of the zebrafish embryo (first row). Scale bars indicate 500 µm.

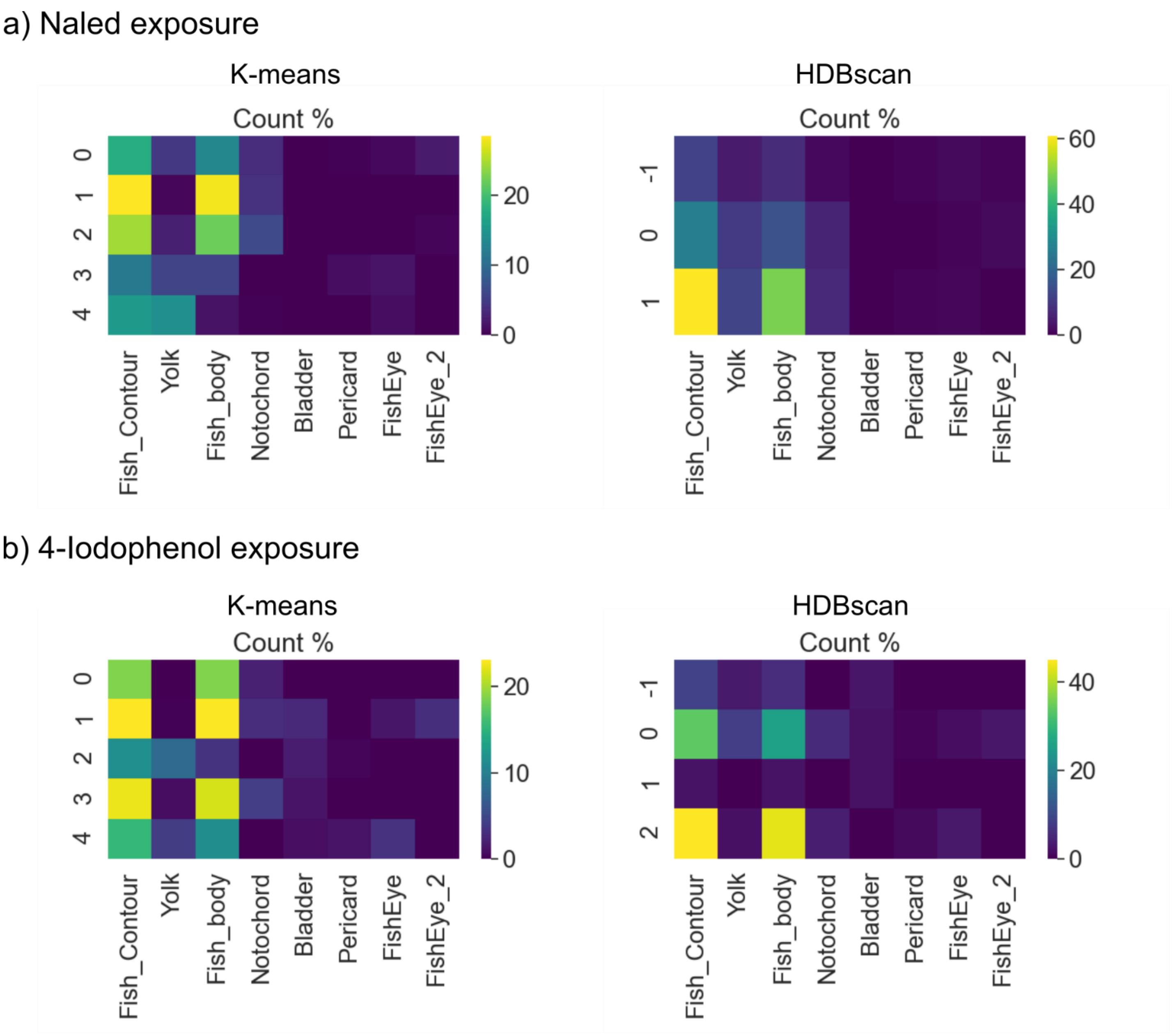

Fig. S5 K-means (numbers of clusters set to 5) and HDBscan (minimum cluster size set to 8 for the naled exposure and 22 for the 4-iodophenol exposure) clustering of ^{12}C and the x- and y-coordinate in embryos exposed a) for 24 h to naled and b) for 96 h to 4-iodophenol. The heatmaps display the percentage of ablation area represented by the cluster and the body part (Count %).

Table S2 Cluster sizes as percentage of total ablation area for the naled exposure and the 4-iodophenol exposure of zebrafish embryos (HDBscan clustering, minimum cluster size set to 8 for the naled exposure and 22 for the 4-iodophenol exposure). The mean intensities of ^{79}Br, ^{127}I, ^{12}C in the clusters, and the quantified bromine and iodine amounts are displayed.

Cluster			Naled exposure			4-Iodophenol exposure		
	Percentage of ablation area (%)	mean intensity ^{79}Br (cps)	mean intensity ^{12}C (cps)	Total Br (ng)	Percentage of ablation area (%)	mean intensity ^{127}I (cps)	mean intensity ^{12}C (cps)	Total I (ng)
-1	12.0	3.82×10^4	3.77×10^5	7.58	9.1	4.20×10^5	2.51×10^5	37.0
0	25.7	4.76×10^4	4.88×10^5	20.7	34.4	1.08×10^5	5.08×10^5	35.2
1	62.3	0	1.44×10^5	0	2.2	1.33×10^4	0	0.27
2	-				54.3	4.33×10^3	0	2.15

Identification of changes over time in the distribution of a xenobiotic in the zebrafish embryo

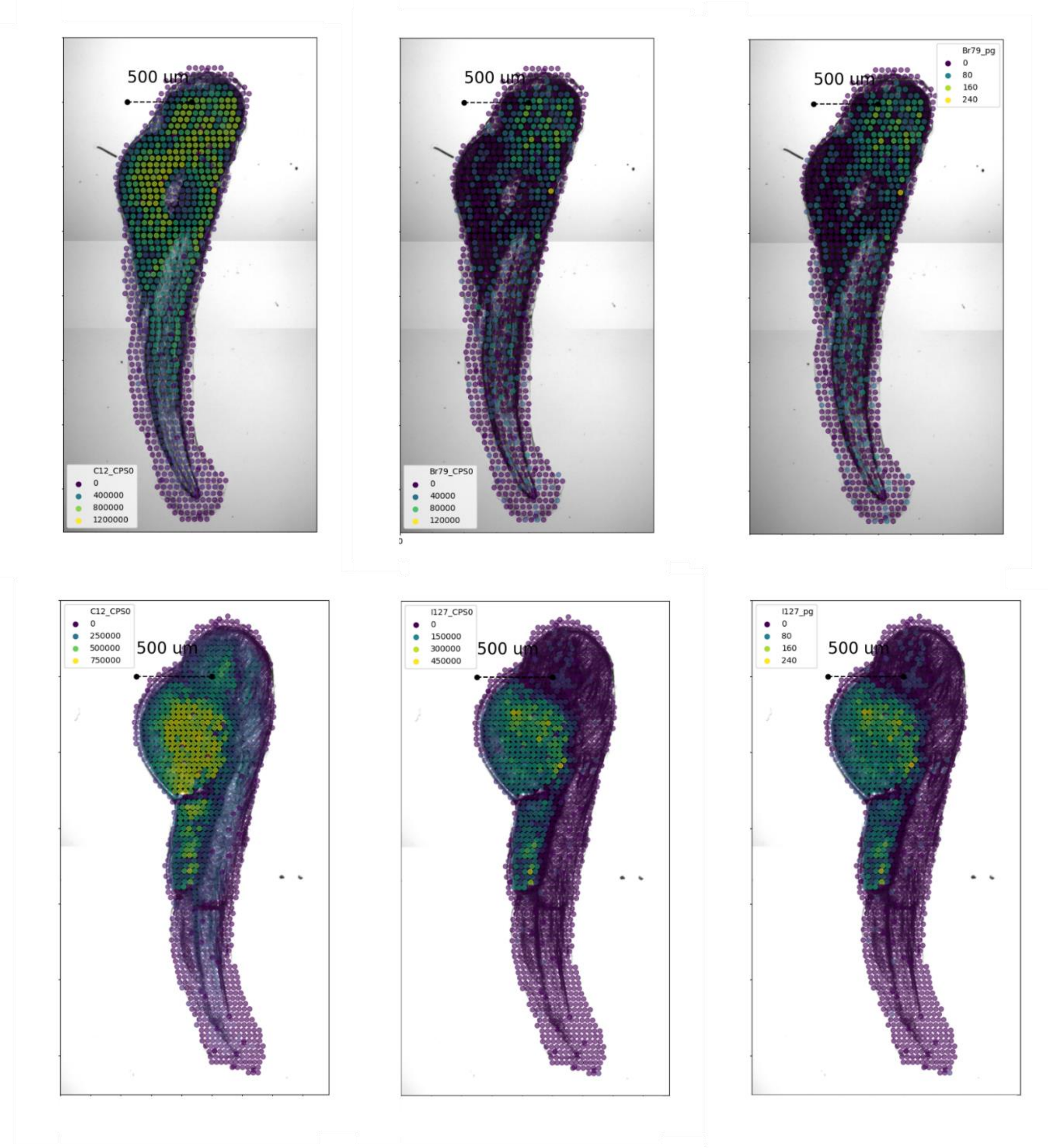

Fig. S6 Intensities of the baseline-corrected carbon (^{12}C), bromine (^{79}Br), and quantified bromine (in pg) LA-ICP-MS signals for the 4 of the zebrafish embryo (first row). Intensities of the baseline-corrected carbon (^{12}C), iodine (^{127}I), and quantified iodine (in pg) LA-ICP-MS signals for the 48 of the zebrafish embryo (first row). Scale bars indicate 500 µm.

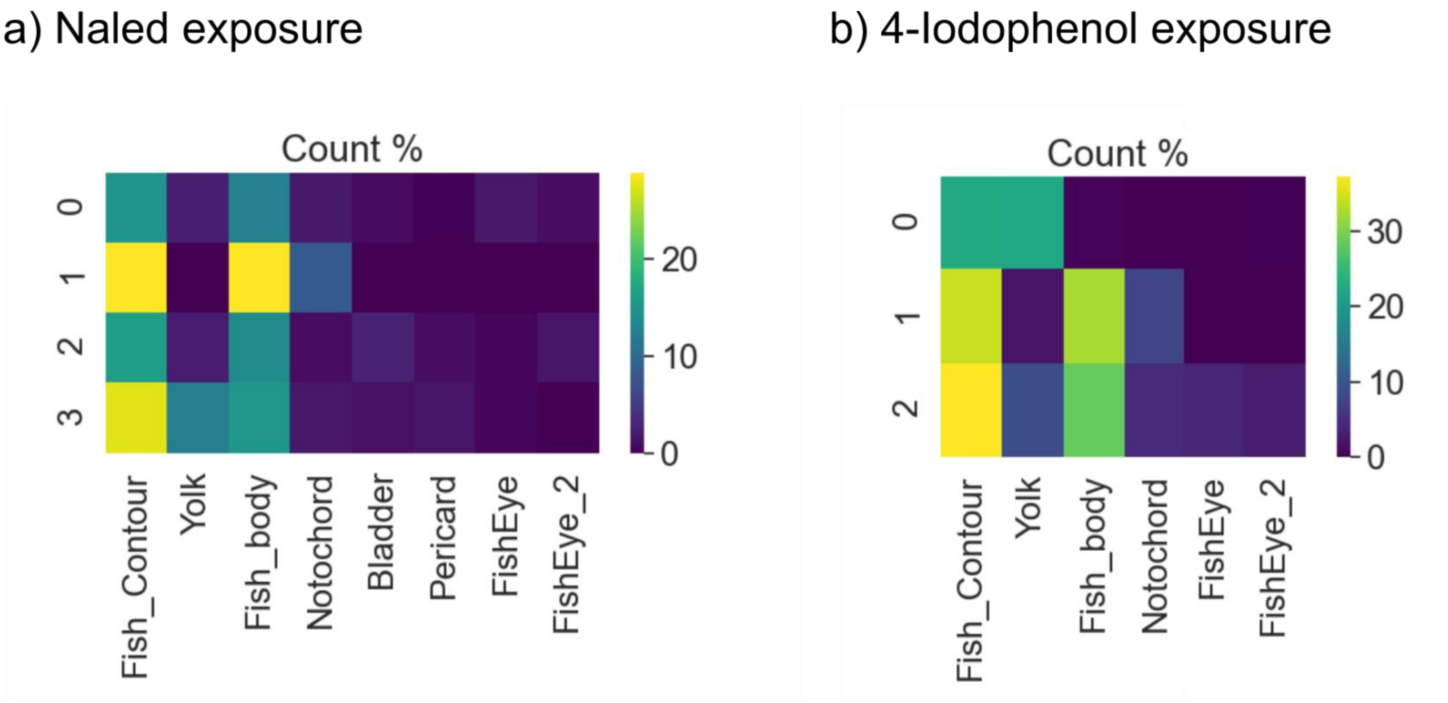

Fig. S7K-means (numbers of clusters set to 4 for the naled exposure and 3 for the 4-iodophenol exposure) clustering of the elemental intensities of ^{12}C, ^{127}I, and the x- and y-coordinate in embryos exposed a) for 4 h to naled and b) for 48 h to 4-iodophenol. The heatmaps display the percentage of ablation area represented by the cluster and the body part (Count %).

Table S3 Cluster sizes as percentage of total ablation area for the naled exposure and the 4-iodophenol exposure of zebrafish embryos (k-means clustering). The mean intensities of ^{79}Br, ^{127}I, ^{12}C in the clusters and the quantified bromine and iodine amounts are displayed.

Cluster	Naled exposure				4-Iodophenol exposure			
	Percentage of ablation area (%)	mean intensity ^{79}Br (cps)	mean intensity ^{12}C (cps)	Total Br (ng)	Percentage of ablation area (%)	mean intensity ^{127}I (cps)	mean intensity ^{12}C (cps)	Total I (ng)
0	15.1	5.34×10^4	7.79×10^5	12.7	22.7	1.71×10^5	4.70×10^5	32.2
1	33.6	6.14×10^3	9.59×10^4	3.93	36.6	1.95×10^3	4.94×10^4	0.71
2	23.8	4.08×10^3	1.33×10^5	1.92	40.7	1.39×10^4	1.37×10^5	5.56
3	27.5	1.41×10^4	5.76×10^5	7.47				

References

1. Teixidó E, Kießling TR, Krupp E, Quevedo C, Muriana A, Scholz S (2019) Automated Morphological Feature Assessment for Zebrafish Embryo Developmental Toxicity Screens. Toxicol Sci 167:438–449 . https://doi.org/10.1093/toxsci/kfy250

2. Brain Map - brain-map.org. https://portal.brain-map.org/. Accessed 21 Apr 2020

3. Kodinariya TM, Makwana PR (2013) Review on Determining of Cluster in K-means Clustering Review on determining number of Cluster in K-Means Clustering. Int J Adv Res Comput Sci Manag Stud 1:90–95
