# Supplementary material for: Effective processing and evaluation of chemical imaging data with respect to morphological features of the zebrafish embryo
Source: Anal Bioanal Chem. 2021 Feb 1;413(6):1675–87. doi: 10.1007/s00216-020-03131-4 (PMC7921040; doi:10.1007/s00216-020-03131-4)

Instructions to reproduce the examples with the software

**Slide 4- 20**	a) Distribution of natural elements in zebrafish embryos and reproducibility between ablations of different individuals – first example
**Slide 21**	a) Distribution of natural elements in zebrafish embryos and reproducibility between ablations of different individuals – second example
**Slide 22**	a) Distribution of natural elements in zebrafish embryos and reproducibility between ablations of different individuals – third example
**Slide 23**	b) Distribution of xenobiotics in the zebrafish embryo – naled exposure
**Slide 24**	b) Distribution of xenobiotics in the zebrafish embryo – 4-iodophenol exposure
**Slide 25**	c) Identification of changes over time in the distribution of a xenobiotic in the zebrafish embryo – naled exposure
**Slide 26**	c) Identification of changes over time in the distribution of a xenobiotic in the zebrafish embryo - 4-iodophenol exposure
**Slide 27**	d) LA-ICP-tof-MS dataset

General

See for an installation guide in https://git.ufz.de/holbrook/fishi-LAICPMS-Imaging-Tool/-/tree/master/test_data

To analyze the data, the software is structured into four tabs which build upon each other:

- Load files
- Transformation and ROIs
- Cluster analysis
- Visualization

The software can be closed by clicking the X in the right upper corner. To load more data tables or images for comparison the *Tools: Table Viewer* or *Tools: Image Viewer* can be used any time during the data analysis. The *Help* menu allows viewing the documentation of the software (*Documentation*), information on the license (*About*), and the version and github (*Read me*). Each created figure may be exported (*Save the figure*) to different image formats (e.g. png, tiff, jpeg). Tables can be easily copied to other files.

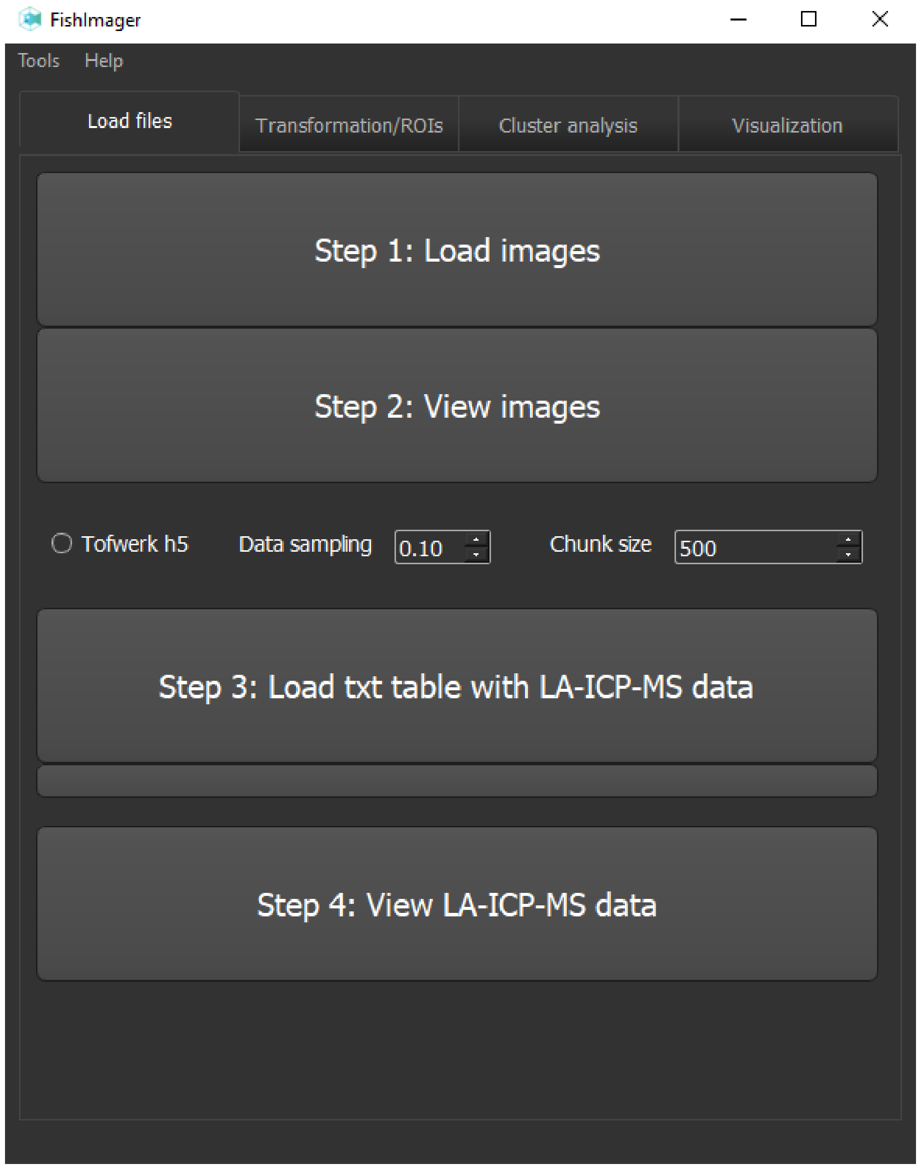

General

The arrow bar at the top of each slide shows at which tab the current instructions belong

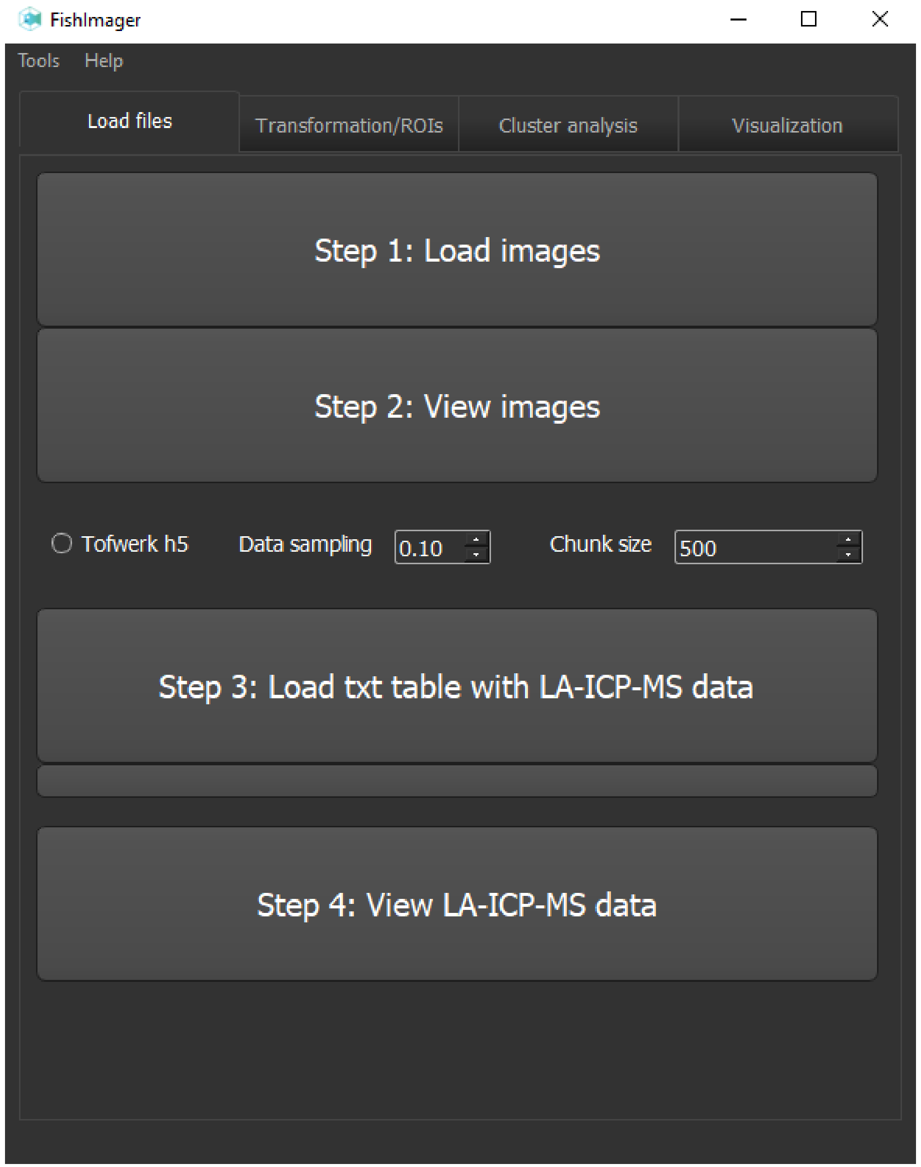

a) Distribution of natural elements in zebrafish embryos and reproducibility between ablations of different individuals – first example

1. Go to the Load files tab.
2. Click *Step 1: Load images*
   - Open in the cloud the folder "Data sets" -> "R&D a).
   - First choose the laser image (Z18NCs_b_1_Img06_Mapped.png).
   - Select cropped Fish inspector image (Z18NCsb1_cropped.png).
   - Select the corresponding coord-file for the laser image (Z18NCs_b_1_Img06.xml).
3. Click *Step 2: View images*
   - Close Figure 1 again.

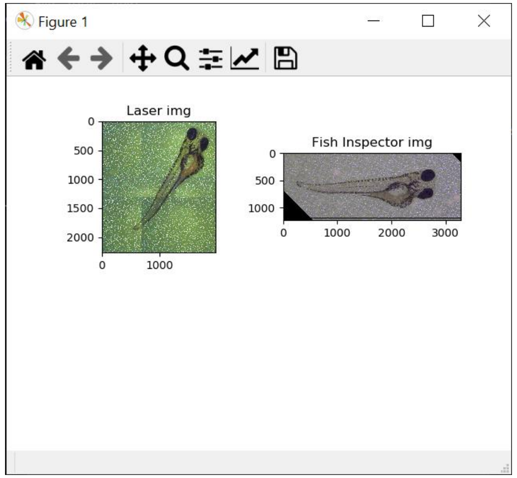

4. Click *Step 3: Load txt table with LA-ICP-MS data*
   - Select the txt file (NC_b_1_tableexport.txt).

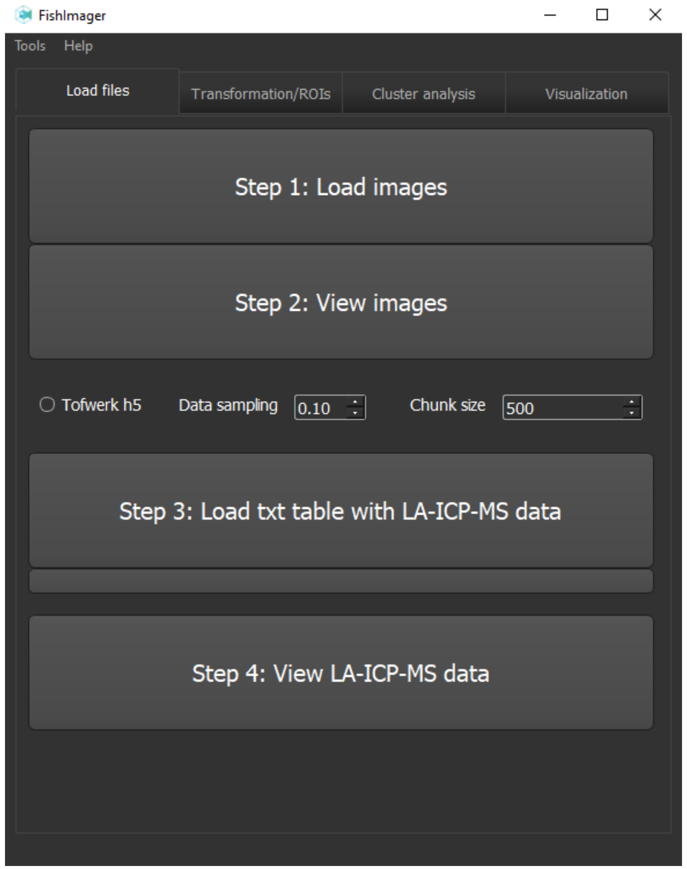

a) Distribution of natural elements in zebrafish embryos and reproducibility between ablations of different individuals – first example

5. You can use the drop-down menu to choose the channel which is displayed for Step 4
   - Select *C12_CPS*

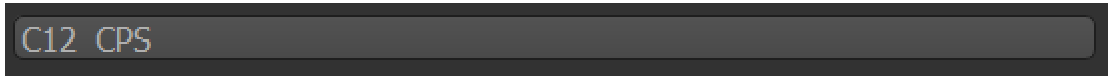

6. Click *Step 4: View LA-ICP-MS data*
   - The imported LA-ICP-MS data are displayed in Figure 1 for the channel selected under 4.
   - You can close Figure 1 again and may select another channel in the drop-down menu from 4, repeat 5.

The figure can also be exported to a png, jpeg, tiff,…

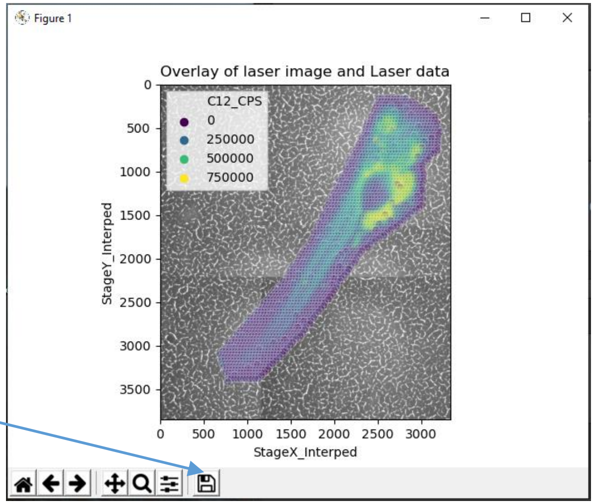

a) Distribution of natural elements in zebrafish embryos and reproducibility between ablations of different individuals – first example

7. Change to the *Transformation and ROIs* tab. Click *Step 1: Choose control points* in order to create an overlay of the laser image and the microscopic image
   - A window appears to inform to close the figure before proceeding with *Step 2*. Click *Okay*.
   - Figure 1 appears showing both images.
   - Choose first one feature on the left image (*Laser img*) and then the corresponding position of this feature with another click on the right image.
   - Perform this for at least 3 control points (here four pints were chosen).
   - The chosen coordinates from the *Laser img* and the corresponding wrapped ones appear in red.
   - **Close Figure 1.**

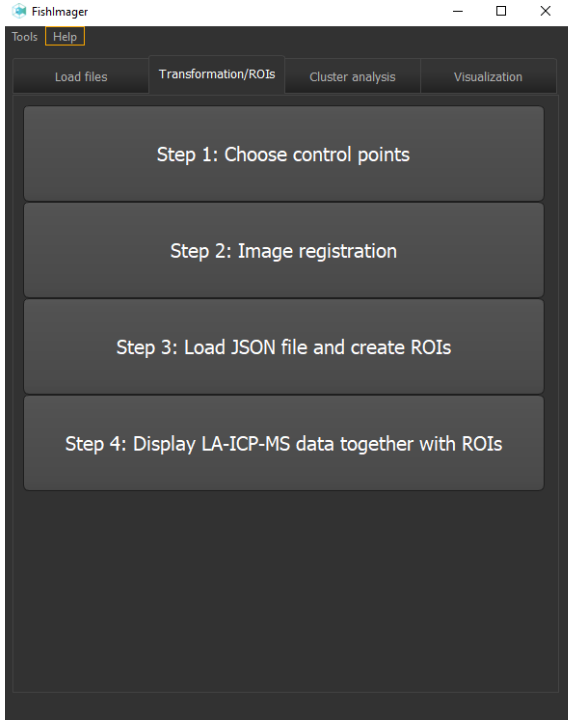

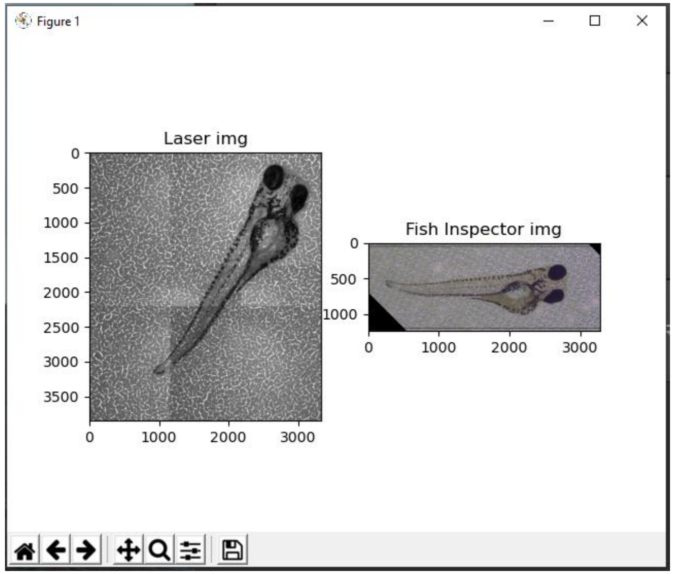

Choose control points

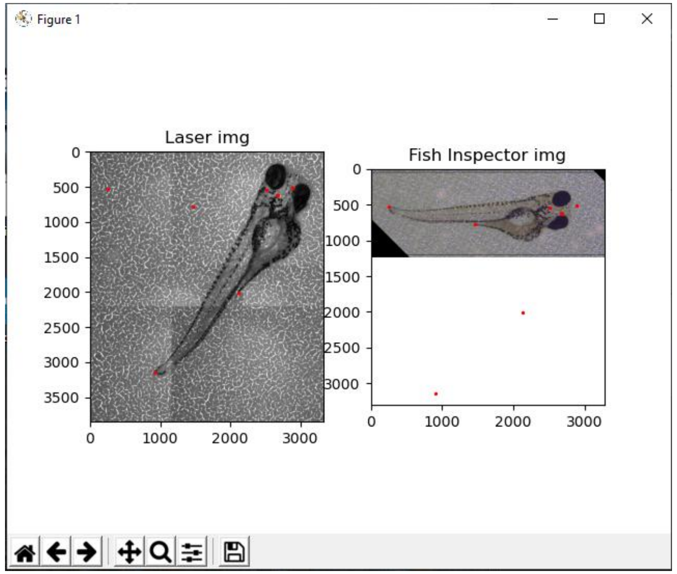

a) Distribution of natural elements in zebrafish embryos and reproducibility between ablations of different individuals – first example

8. Click *Step 2: Image registration* to see the overlay in the appearing window "Figure 1"
    - A window appears to inform to close the figure before proceeding with *Step 3*. Click *Okay*.
    - **Close Figure 1.**
    - A window appears to inform whether the transformation was successful. Click *Okay*.

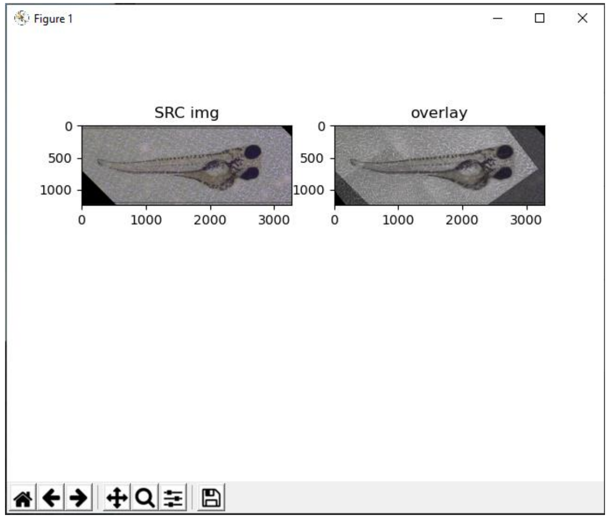

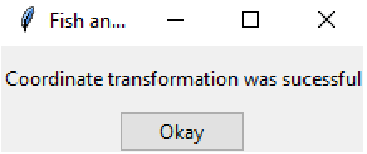

9. The file with the shapes of the biological body compartments from the FishInspector software is loaded with *Step 3: Load JSON file and create ROIs* (Z18NCsb1_cropped__SHAPES.json)
    - The window "Figure 1" opens and displays the outlines of the biological compartment on
      the transformed laser image ("Laser img wrapped") and
      the microscopic image ("Fish Inspector img").
    - Close Figure 1.

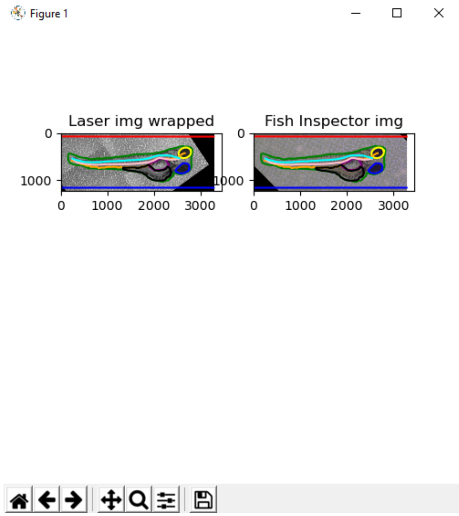

a) Distribution of natural elements in zebrafish embryos and reproducibility between ablations of different individuals – first example

10. Click *Step 4: Display LA-ICP-MS data together with ROIs*

- The window “Figure 1“ opens and displays LA-ICP-MS data on the transformed laser image with the biological compartments.
- The notochord consists out of two lines in the FishInspector software. Here the area is calculated between the two lines and all coutns belonging to this area are colored white.
- Close Figure 1.

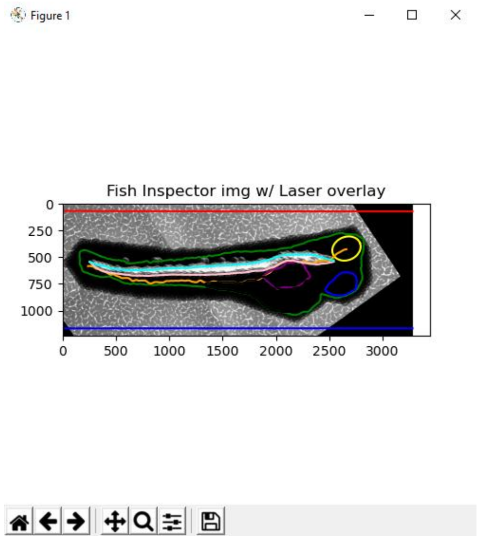

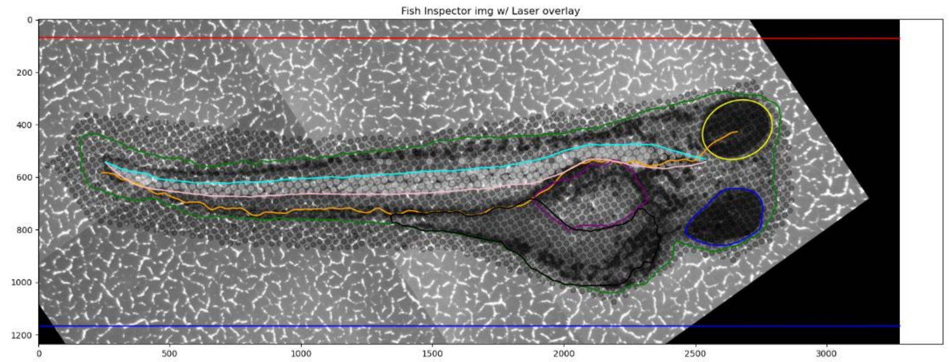

a) Distribution of natural elements in zebrafish embryos and reproducibility between ablations of different individuals – first example

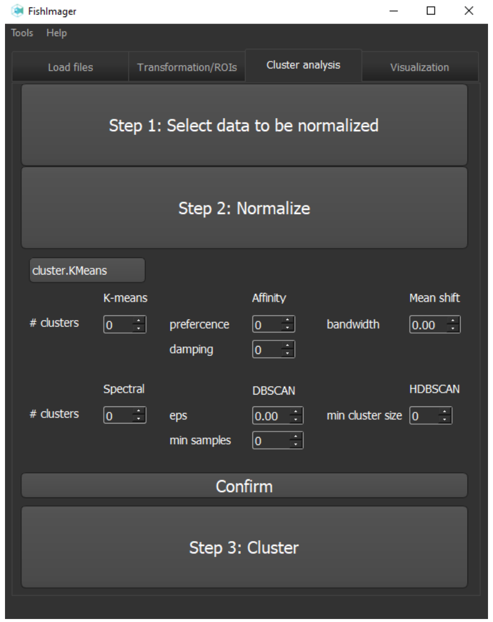

11. Change to the *Cluster analysis* tab. Click *Step 1: Select data to be normalized.*
    - Open the LA-ICP-MS data table (NC_b_1_tableexport.txt).
    - This opens the table with the measured elements and the coordinates.
    - Delete the columns which should **not** be included in the cluster analysis by right click on the respective column head.
    - The remaining columns should be “C12_CPS, StageX_Interped, StageY_Interped“.
    - Close the table app.

12. Click *Step 2: Normalize*.
    - In order to change the selection, go back to 4. Repeat 11. and 12.

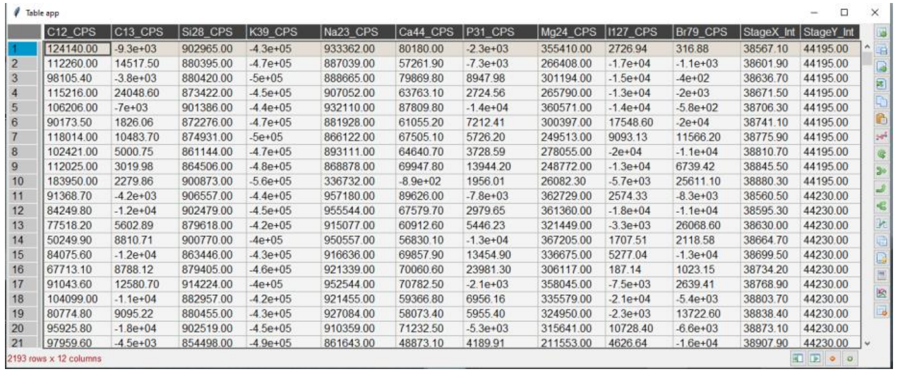

Table app

	C12_CPS	C13_CPS	Si28_CPS	K39_CPS	Na23_CPS	Ca44_CPS	P31_CPS	Mg24_CPS	I127_CPS	Br79_CPS	StageX_Int	StageY_Int
1	124140.00	-9.3e+03	902965.00	-4.3e+05	933362.00	80180.00	-2.3e+03	355410.00	2726.94	316.88	38567.10	44195.00
2	112260.00	14517.50	880395.00	-4.7e+05	887039.00	57261.90	-7.3e+03	266408.00	-1.7e+04	-1.1e+03	38601.90	44195.00
3	98105.40	-3.8e+03	880420.00	-5e+05	888665.00	79869.80	8947.98	301194.00	-1.5e+04	-4e+02	38636.70	44195.00
4	115216.00	24048.60	873422.00	-4.5e+05	907052.00	63763.10	2724.56	265790.00	-1.3e+04	-2e+03	38671.50	44195.00
5	106206.00	-7e+03	901386.00	-4.4e+05	932110.00	87809.80	-1.4e+04	360571.00	-1.4e+04	-5.8e+02	38706.30	44195.00
6	90173.50	1826.06	872276.00	-4.7e+05	881928.00	61055.20	7212.41	300397.00	17548.60	-2e+04	38741.10	44195.00
7	118014.00	10483.70	874931.00	-5e+05	866122.00	67505.10	5726.20	249513.00	9093.13	11566.20	38775.90	44195.00
8	102421.00	5000.75	861144.00	-4.7e+05	893111.00	64640.70	3728.59	278055.00	-2e+04	-1.1e+04	38810.70	44195.00
9	112025.00	3019.98	864506.00	-4.8e+05	868878.00	69947.80	13944.20	248772.00	-1.3e+04	6739.42	38845.50	44195.00
10	183950.00	2279.86	900873.00	-5.6e+05	336732.00	-8.9e+02	1956.01	26082.30	-5.7e+03	25611.10	38880.30	44195.00
11	91368.70	-4.2e+03	906557.00	-4.4e+05	957180.00	89626.00	-7.8e+03	362729.00	2574.33	-8.3e+03	38560.50	44230.00
12	84249.80	-1.2e+04	902479.00	-4.5e+05	955544.00	67579.70	2979.65	361360.00	-1.8e+04	-1.1e+04	38595.30	44230.00
13	77518.20	5602.89	879618.00	-4.2e+05	915077.00	60912.60	5446.23	321449.00	-3.3e+03	26068.60	38630.00	44230.00
14	50249.90	8810.71	900770.00	-4e+05	950557.00	56830.10	-1.3e+04	367205.00	1707.51	2118.58	38664.70	44230.00
15	84075.60	-1.2e+04	863446.00	-4.3e+05	916636.00	69857.90	13454.90	336675.00	5277.04	-1.3e+04	38699.50	44230.00
16	67713.10	8788.12	879405.00	-4.6e+05	921339.00	70060.60	23981.30	306117.00	187.14	1023.15	38734.20	44230.00
17	91043.60	12580.70	914224.00	-4e+05	952544.00	70782.50	-2.1e+03	358045.00	-7.5e+03	2639.41	38768.90	44230.00
18	104099.00	-1.1e+04	882957.00	-4.2e+05	921455.00	59366.80	6956.16	335579.00	-2.1e+04	-5.4e+03	38803.70	44230.00
19	80774.80	9095.22	880455.00	-4.3e+05	927084.00	58073.40	5955.40	324950.00	-2.3e+03	13722.60	38838.40	44230.00
20	95925.80	-1.8e+04	902519.00	-4.5e+05	910359.00	71232.50	-5.3e+03	315641.00	10728.40	-6.6e+03	38873.10	44230.00
21	97959.60	-4.5e+03	854498.00	-4.9e+05	861643.00	48873.10	4189.91	211553.00	4626.64	-1.6e+04	38907.90	44230.00

2193 rows x 12 columns

a) Distribution of natural elements in zebrafish embryos and reproducibility between ablations of different individuals – first example

13. The cluster algorithm can be chosen at this point.
    - Choose *cluster.Kmeans* and set the number of clusters *(# cluster)* to 4.

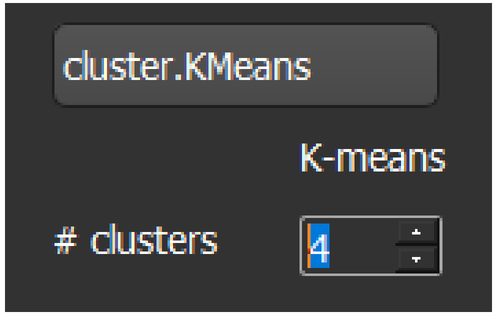

14. Click *Confirm*. In the case of the Kmeans clustering, a graphic with the elbow method to choose the optimal k for k=1-9 appears
    - Instruction number 12 may be repeated if the numbers of cluster should be changed.
    - Close Figure 1.

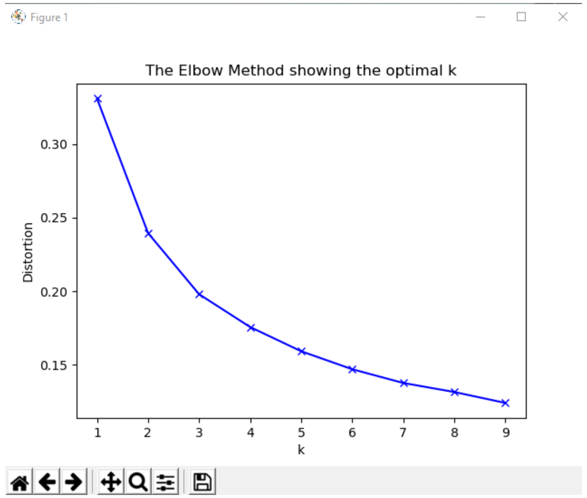

a) Distribution of natural elements in zebrafish embryos and reproducibility between ablations of different individuals – first example

15. Click *Step 3: Cluster*.
   - The clustered LA-ICP-MS data appear in Figure 1.
   - Close Figure 1.

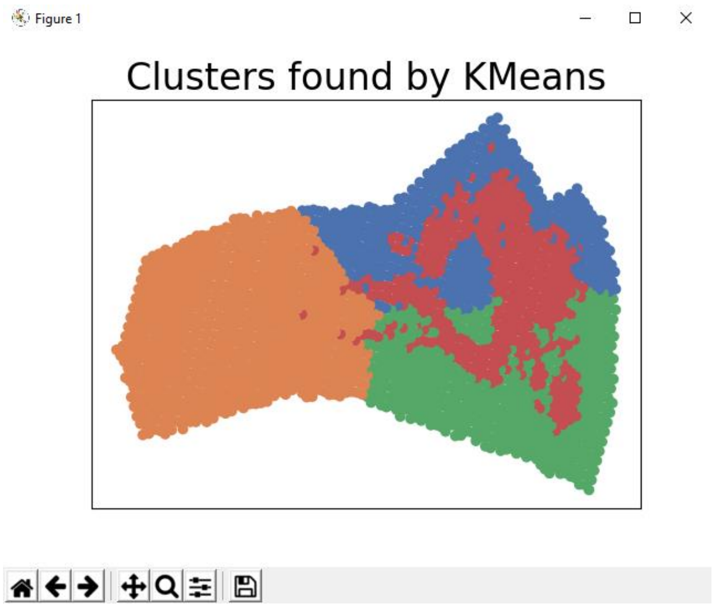

a) Distribution of natural elements in zebrafish embryos and reproducibility between ablations of different individuals – first example

16. Change to the *Visualization* tab. Click *Step 1: Visualization*.
    - The Figure 1 (*ROI Cluster Image overlay*) shows the color-coded, clustered LA-ICP-MS data with the ROIs.
    - Close Figure 1.

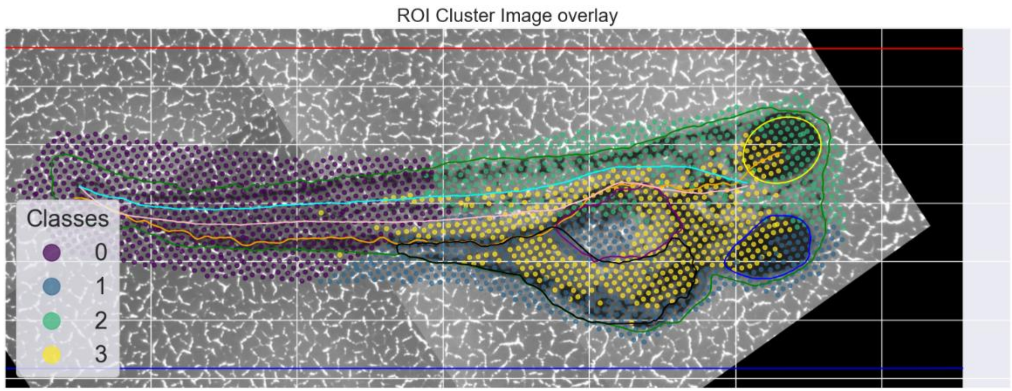

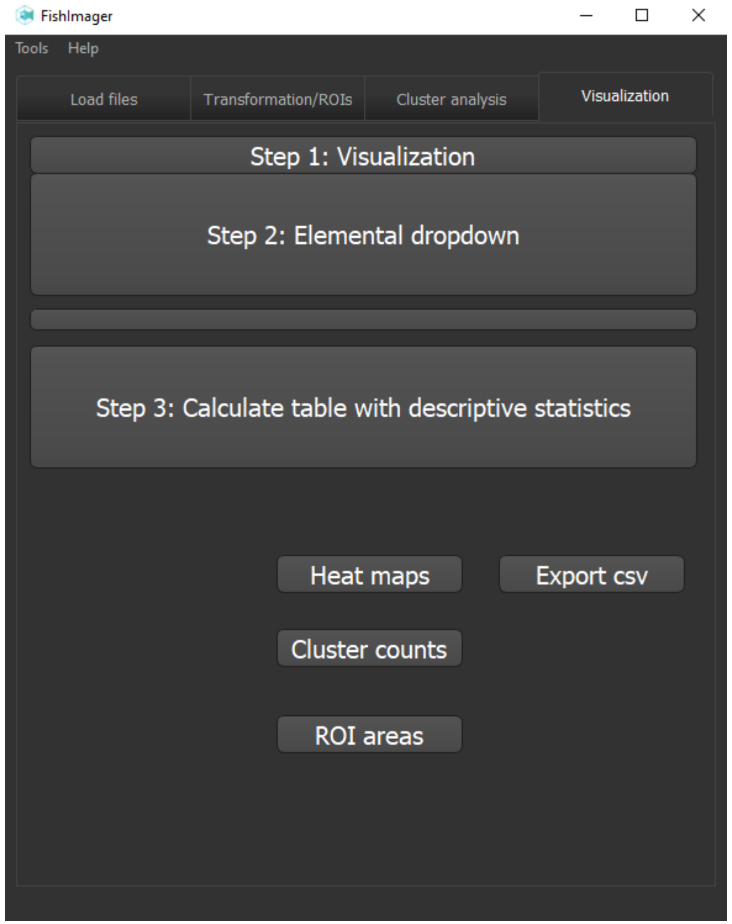

a) Distribution of natural elements in zebrafish embryos and reproducibility between ablations of different individuals – first example

17. Click *Step 2: Elemental dropdown*.
    - A warning message advises to choose an elemental channel in the following drop-down menu. Click *OK* to remove the message.

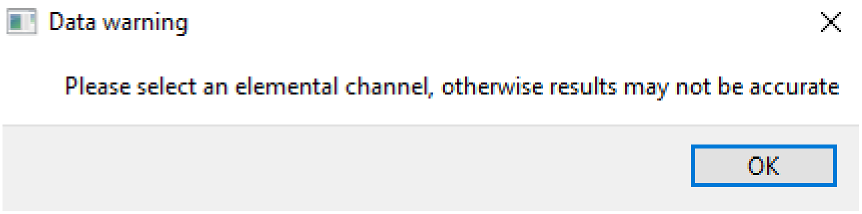

18. Choose an elemental channel in the drop-down menu which had been included for the cluster analysis (C12_CPS) to investigate.

a) Distribution of natural elements in zebrafish embryos and reproducibility between ablations of different individuals – first example

19. Click *Step 3: Calculate table with descriptive statistics*.
    - A table opens with the descriptive statistics of the chosen elemental channel for each cluster.
    - The sum, mean, standard deviation, median, variance, minimum and maximum is displayed for the channel (P31_CPS) for each cluster.
    - Close the *Table app*.

Table app

	sum	mean	std	median	var	min	max
1	54623126.20	135878.40	78057.50	118564.00	6092974200.20	-5.8e+03	357177.00
2	106724520.00	131758.70	71876.10	99130.60	5166168733.10	-9e+03	353414.00
3	103197975.70	199996.10	102729.90	193284.00	10553437576.70	3014.90	454233.00
4	229745657.00	494076.70	111454.80	489367.00	12422168074.30	292584.00	736366.00

4 rows x 7 columns

a) Distribution of natural elements in zebrafish embryos and reproducibility between ablations of different individuals – first example

20. Click *Heatmaps*.

- Five heatmaps appear relating to the elemental channel selected under 18.
- In case the margins of the heatmaps need to be adjusted, use the *Configure subplots* button (see arrow).
- The calculation of the different heatmaps are explained on the following slides.
- The data for the calculation of the heatmaps can be exported as csv-file (*Export csv*).

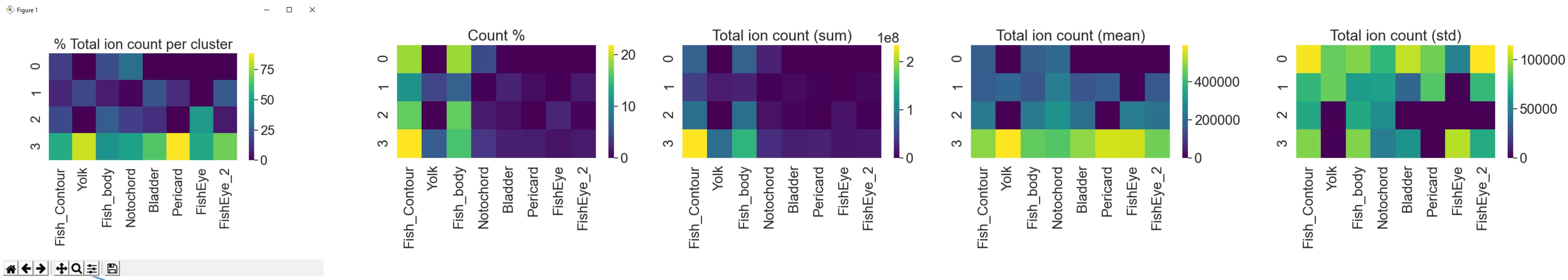

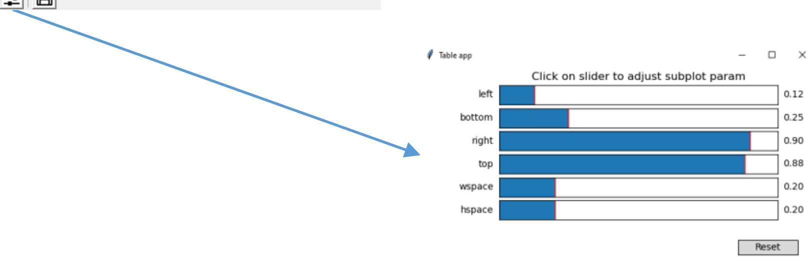

a) Distribution of natural elements in zebrafish embryos and reproducibility between ablations of different individuals – first example

Total ion count (sum) – Figure 3

- This heatmap displays the sum of the elemental channel (selected under 18.) per cluster and body part.
- E.g. the total intensity in the bladder is partly represented by cluster 2 and 3: both have a total intensity of around 0.25e8.
- E.g. the total intensity of the area overlapping of cluster 3 and the yolk is circa 1e8, of cluster 0 and the yolk 0.25e8.

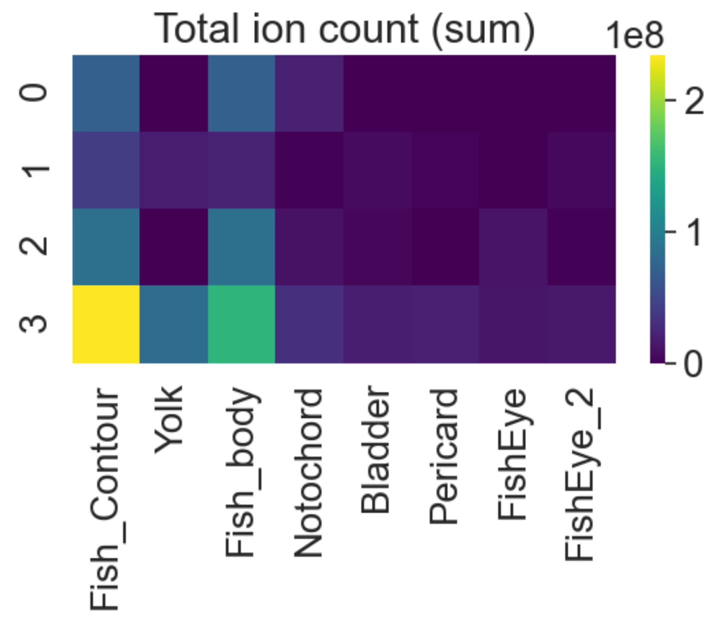

a) Distribution of natural elements in zebrafish embryos and reproducibility between ablations of different individuals – first example

% Total ion count per cluster – Figure 1

- This heatmap displays the sum of the elemental channel (selected under 18.) per cluster normalized to each body part as percentage.
- I.e. the sum of the elemental channel in each cluster per body part sums up to 100%, respectively.
- The heatmap represents the same information as *Total ion count (sum)* but in percentage per each body compartment.
- E.g. the total intensity in the bladder is around 60% represented by cluster 3 and between 15 and 25% by cluster 1 and 2.
- E.g. circa 70% of the total intensity in the yolk is represented by cluster 3, 30% by cluster 0.

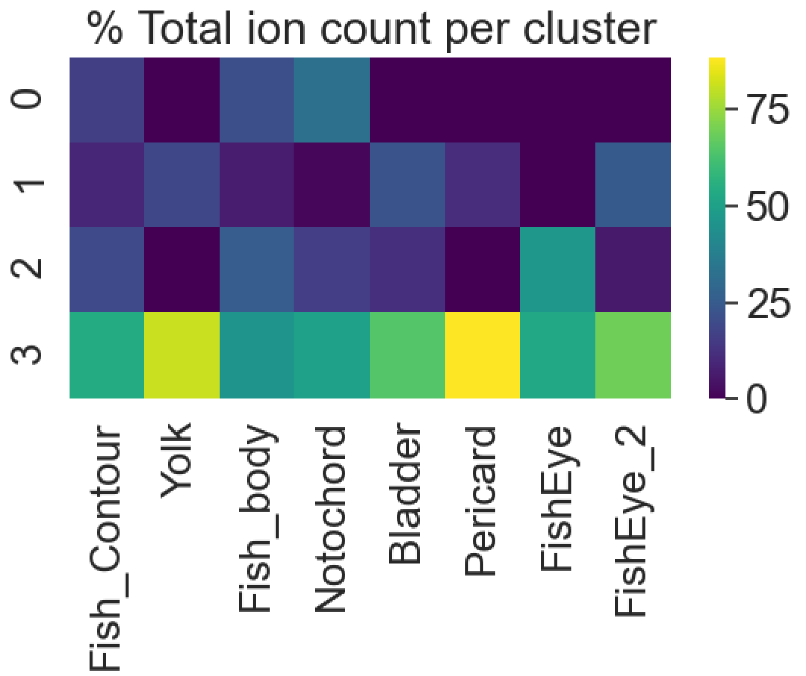

a) Distribution of natural elements in zebrafish embryos and reproducibility between ablations of different individuals – first example

Total ion count (mean) – Figure 4

- This heatmap displays the mean of the elemental channel (selected under 18.) per cluster and per body compartment, respectively.
- E.g. the mean intensity of C12_CPS in the area shared by the bladder and cluster 3 is circa 4.5e6, the mean intensity in the area shared by the bladder and cluster 2 is circa 2.5e6, by the bladder and cluster 0 1.5e6.
- E.g. The mean intensity in the ROI yolk represented by cluster 3 is circa 6e6, by cluster 0 1.5e6.
- The corresponding standard deviation (std) is also visualized in a heatmeap (*Total ion count (std)*).

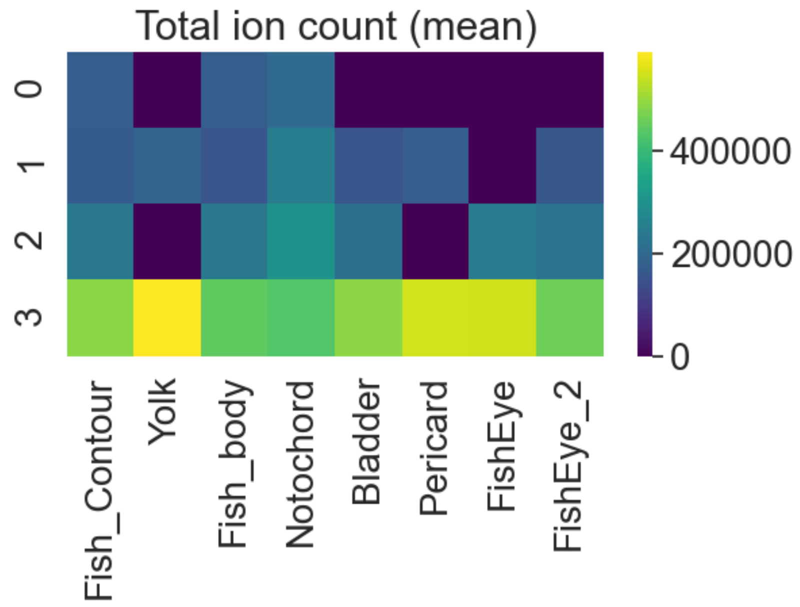

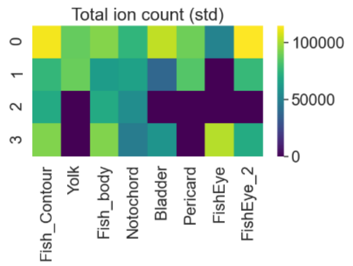

a) Distribution of natural elements in zebrafish embryos and reproducibility between ablations of different individuals – first example

Count %

- This heatmap displays the number of counts (pixels) of the LA-ICP-MS data per cluster normalized to the shape of the data matrix as percentage.
- E.g. around 4% of the counts in cluster 2 are represented by the body compartment bladder, 10% of cluster 2 is represented by the body compartment yolk.

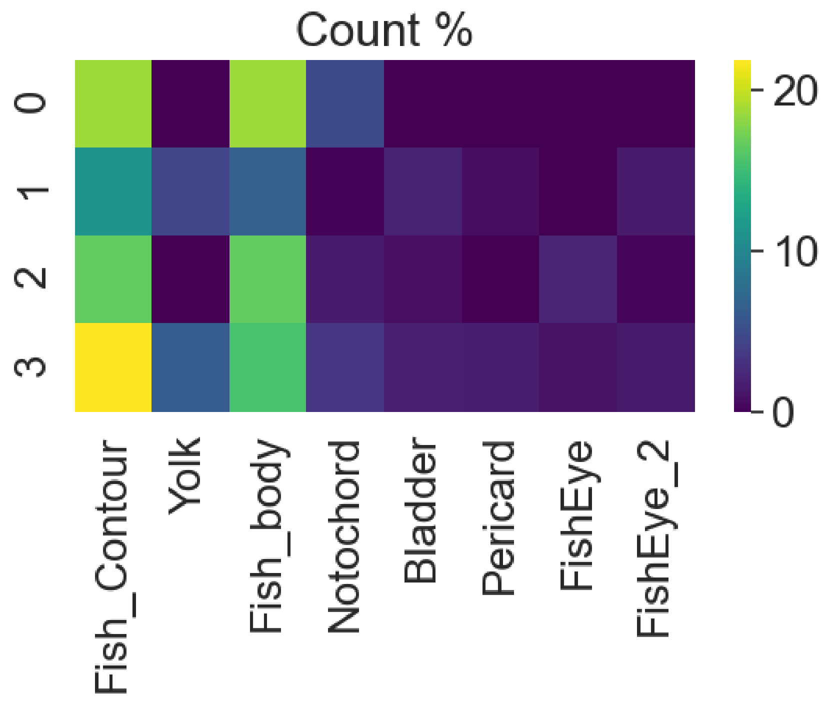

a) Distribution of natural elements in zebrafish embryos and reproducibility between ablations of different individuals – first example

21. Click *Cluster counts*.
    - The table show the total counts in each cluster and the percentage of the total measured counts.
    - Close the Table app.
22. Click *ROI areas*.
    - The table displays the percentage of each body compartment on the whole Fish Contour.

cluster_labels	Total counts	percent total
0	806	36.75331
1	402	18.33105
2	503	22.93662
3	482	21.97902

Table app

	strings	Area %
1	Fish_Contour	100.00
2	FishEye	4.70
3	Bladder	7.34
4	Yolk	16.00
5	FishEye_2	4.60
6	Fish_body	84.00
7	Notochord	25.21
8	Pericard	3.61

8 rows x 2 columns

a) Distribution of natural elements in zebrafish embryos and reproducibility between ablations of different individuals – second example

- First choose the laser image (Z18NCs_b_2_Img05_Mapped.png).
- Select cropped Fish inspector image (Z18NCsb2__cropped.png).
- Select the corresponding coord-file for the laser image (Z18NCs_b_Img05.xml).
- NCs_b_2_tableexport.txt
- Z18NCsb2_cropped__SHAPES.json
- Cluster 12C_CPS, X_Stage_Interped, Y_Interped

a) Distribution of natural elements in zebrafish embryos and reproducibility between ablations of different individuals – third example

- First choose the laser image (Z18NCs_b_Img04_Mapped.png).
- Select cropped Fish inspector image (Z18NCsb3_cropped.png).
- Select the corresponding coord-file for the laser image (Z18NCs_b_Img04.xml).
- NCs_b_3_tableexport.txt
- Z18NCsb3_cropped__SHAPES.json
- Cluster 12C_CPS, X_Stage_Interped, Y_Interped

b) Distribution of xenobiotics in the zebrafish embryo – naled exposure

- First choose the laser image (Z18A12u3A21u2_Img06_Mapped.png).
- Select cropped Fish inspector image (Z18A2_1_cropped.png).
- Select the corresponding coord-file for the laser image (Z18A12u3A21u2_Img06.xml).
- Z18A2_1_table_iolite_output.txt
- Z18A2_1_cropped__SHAPES.json
- Cluster 12C_CPS0, 79Br_CPS0, X_Stage_Interped, Y_Interped

b) Distribution of xenobiotics in the zebrafish embryo – 4-iodophenol exposure

- First choose the laser image (Z19D1_4_Img06_Mapped.png).
- Select cropped Fish inspector image (Z19D1_4_cropped.png).
- Select the corresponding coord-file for the laser image (Z19D1_4_Img06.xml).
- Z19D1_4_TableExport.txt
- Z19D1_4_cropped__SHAPES.json
- Cluster 12C_CPS0, 127I_CPS0, X_Stage_Interped, Y_Interped

c) Identification of changes over time in the distribution of a xenobiotic in the zebrafish embryo – naled exposure

- First choose the laser image (Z18C11u2C23Img05_Mapped.png).
- Select cropped Fish inspector image (Z18C2_4_cropped.png).
- Select the corresponding coord-file for the laser image (Z18C11u2C23Img05.xml).
- Z18C2_4_iolite_output.txt
- Z18C2_4_cropped__SHAPES.json
- Cluster 12C_CPS0, 127Ir_CPS0, X_Stage_Interped, Y_Interped

d) Identification of changes over time in the distribution of a xenobiotic in the zebrafish embryo – 4-iodophenol exposure

- First choose the laser image (Z19B1_2_Img09_Mapped.png).
- Select cropped Fish inspector image (Z19B1_2_cropped.png).
- Select the corresponding coord-file for the laser image (Z19B1_2_Img09.xml).
- Z19B1_2_TableExport.xml
- Z19B1_2_cropped__SHAPES.json
- Cluster 12C_CPS0, 127Ir_CPS0, X_Stage_Interped, Y_Interped

d) LA-ICP-tof-MS dataset

- First choose the laser image (Z34D3_3_Img01_Mapped.png).
- Select cropped Fish inspector image (Z34D3_3_cropped.png).
- Select the corresponding coord-file for the laser image (Z34D3_3_Img01.xml).

d) LA-ICP-tof-MS dataset

- Go to the Load files tab.
- Check the Tofwerk h5 button.
- Choose 0.5 for data sampling and 500 for chunk size.
- Click *Step 3.*
- The following message appears, Click *OK.*

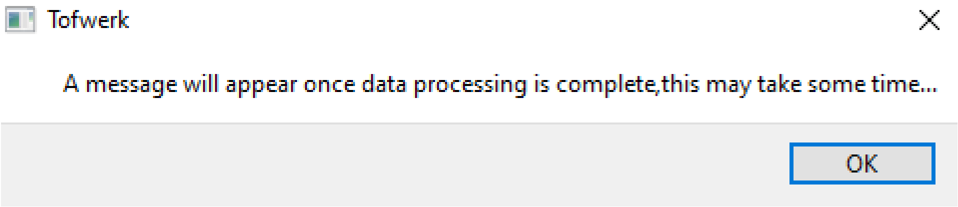

- Select the following file Laser_2020.05.18.13h45m30s_Z34D3_4_50x50_50umsec_50hz_2J.h5
- After a short time, FishI shows that the data processing is complete.

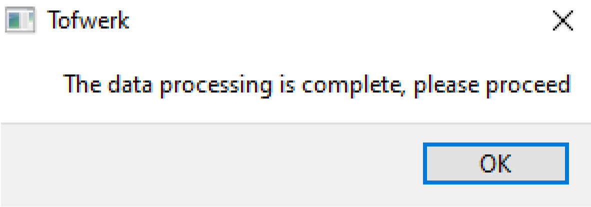

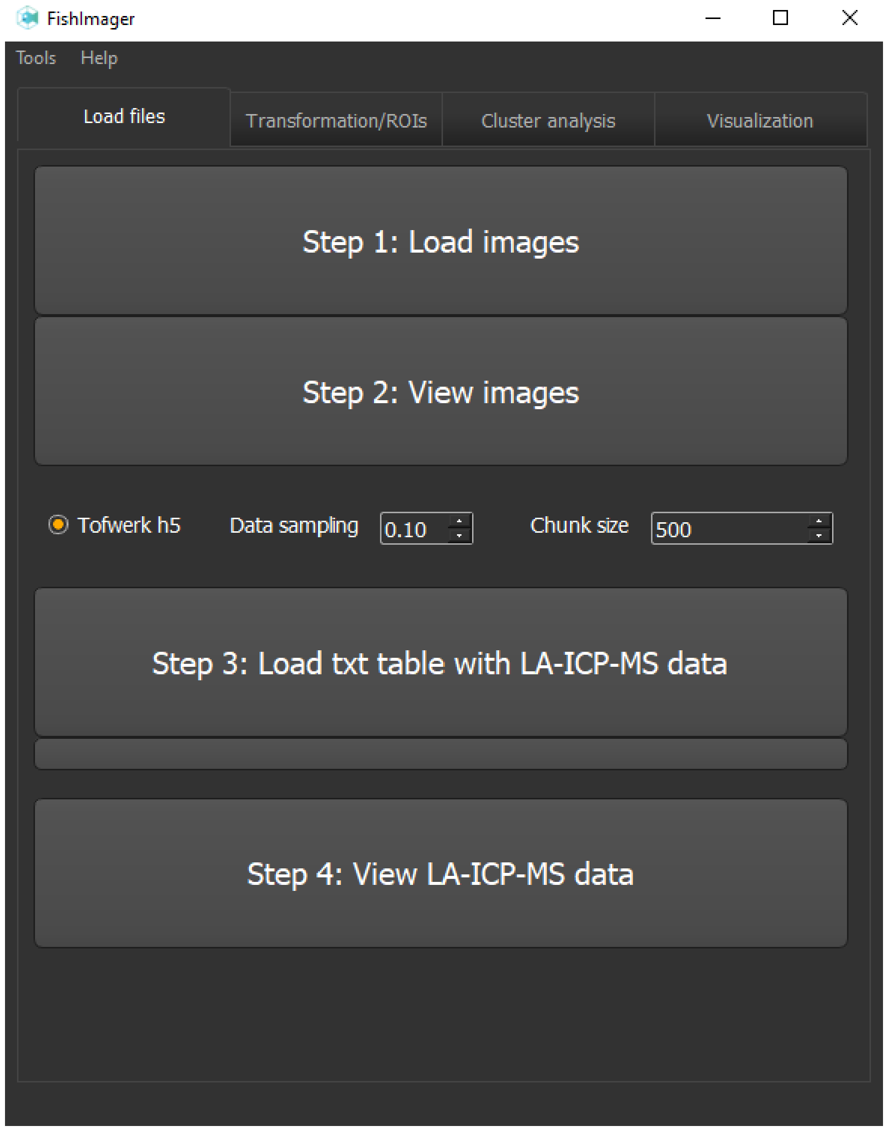

d) LA-ICP-tof-MS dataset

- You can proceed now as with the other examples.
- Corresponding FishInspector file
  - Z34D3_3_cropped__SHAPES.json
- For tof-data visualization, please disregard the plot appearing in *Cluster analysis*, *Step 3: Cluster*

  -> the data which are displayed are not in the correct coordinate system
- For normalization in the *Cluster analysis* tab choose, e.g. 12C, 13C, 14N, 15N, 23Na, 31P, 15N, 32S, 33S, 34S, 35Cl, 36S, 37Cl, 39K, 40Ar, 40Ca, 41K, 42Ca, 43Ca, 44Ca, 46Ca, 48Ca, 37Cl, 127I

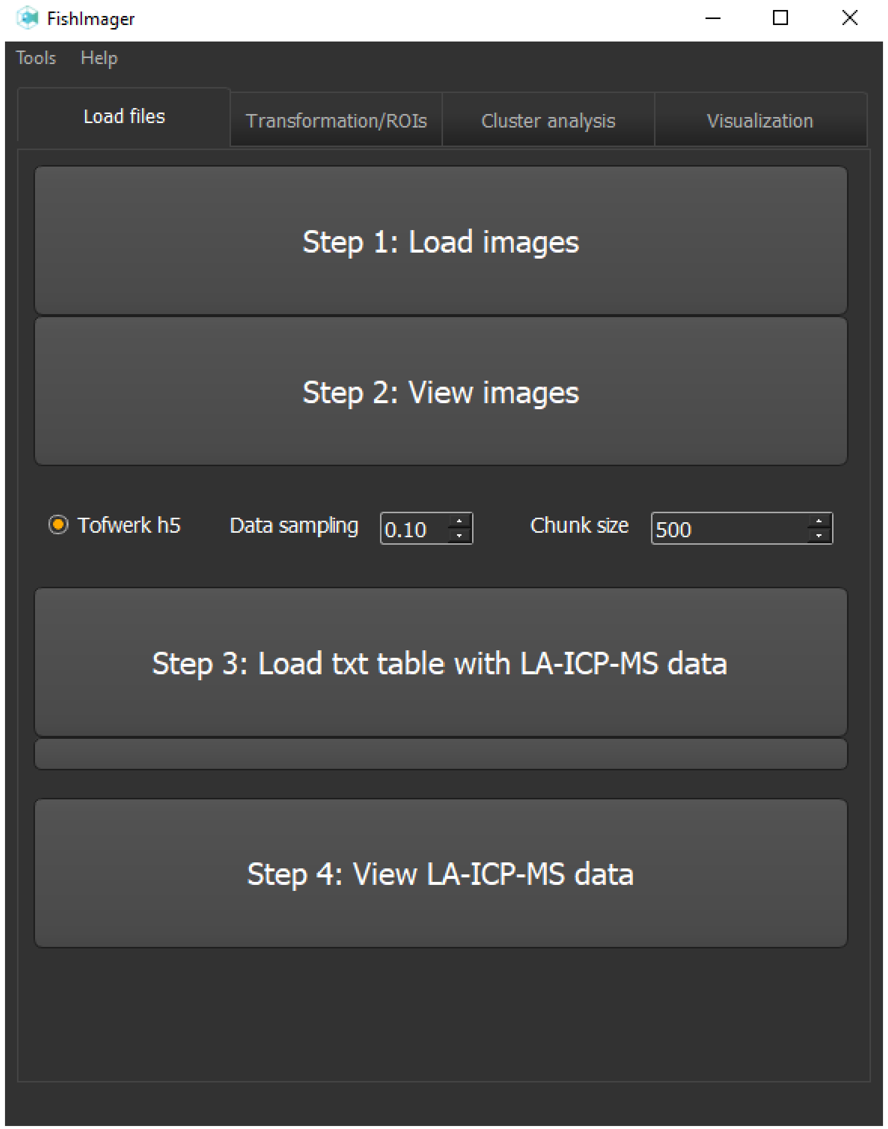

Supplement: Supplementary file 2 — (Supplementary_information_2.pdf) Tutorial for using FishImager and reproducing the examples in the manuscript. (PDF 1335 kb) [file 216_2020_3131_MOESM2_ESM.pdf]
